# Isomeric Speciation of Bisbenzoxazine Intermediates by Ion Spectroscopy and Ion Mobility Mass Spectrometry

**DOI:** 10.1021/acsomega.4c06205

**Published:** 2024-09-18

**Authors:** Francisco W. M. Ribeiro, Danilo Silva-Oliveira, Gustavo Cervi, Eduardo D. Koyanagui, Thiago C. Correra

**Affiliations:** Department of Fundamental Chemistry, Institute of Chemistry, University of São Paulo, Av. Prof. Lineu Prestes, 748, Cidade Universitária, São Paulo, São Paulo 05508-000, Brazil

## Abstract

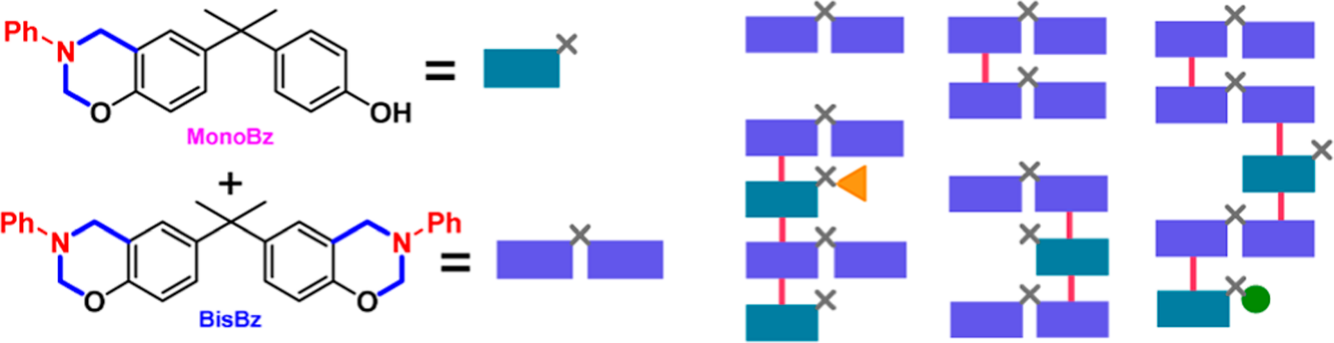

Bisbenzoxazines (**BisBz**) are a relevant model for the diverse bifunctional benzoxazines that are used to increase the polybenzoxazines cross-linking extensions and modulate the final resin properties for various usages. The presence of side products and intermediates during monomer formation can influence the resin characteristics by inducing chain termination and ramifications, affecting the polymerization and cure processes. This work investigated the diverse isomeric intermediates and side products that are present during the **BisBz** formation from bisphenol A, aniline, and formaldehyde by ion mobility coupled to *tandem* mass spectrometry (MS/MS) and ion spectroscopy techniques. The species detected in this work suggest that these multifunctional phenols open diverse concurrent reaction pathways based on two main reactive steps: (i) the imine/iminium phenol attack to form a phenylamino intermediate and (ii) the formaldehyde attack followed by dehydration to form the oxazine ring. The species observed also support previous studies of the benzoxazine formation mechanism and showcase the application of advanced analytical techniques in studying complex chemical systems.

## Introduction

Bisbenzoxazines (**BisBz**) are cyclic compounds that have two or more oxazine rings in their structure.^1−3^ The use of these compounds as precursors of polybenzoxazine (**PBz**) resins is widespread in aerospace, pharmaceutical, and electronic industries where they have been employed as flame retardant agents, adhesive coatings, and drug delivery manifolds, between other applications.^4,5^

The versatility of these polymeric species are reported to be closely correlated to the structure of the initial monomers as minor structural changes have great influence on the final characteristics and performance of these resins.^6−9^

For instance, Liu and Ishida^10^ reported that polymers derived from 2,2′-, 2,4′-, and 4,4′-substituted benzoxazine monomers based on bisphenol F and aniline present distinct thermal and mechanical properties. The 2,2′-substituted benzoxazine showed higher thermal stability and a cross-link density approximately 20 and 7.5 times greater than the 2,4′- and 4,4′-substituted **PBz**, respectively.

Liu and co-workers studied different isomeric benzoxazine resins of natural origin based on carvacrol (3-isopropyl-5-methylphenol) and thymol (2-isopropyl-5-methylphenol) as phenolic compounds and furfurylamine as the primary amine.^7^ They reported that carvacrol-based benzoxazines showed signs of impurities originated from the carvacrol reaction with the benzoxazine monomer that were not observed with thymol because of distinct steric effects between these phenols.

**Figure 1:**
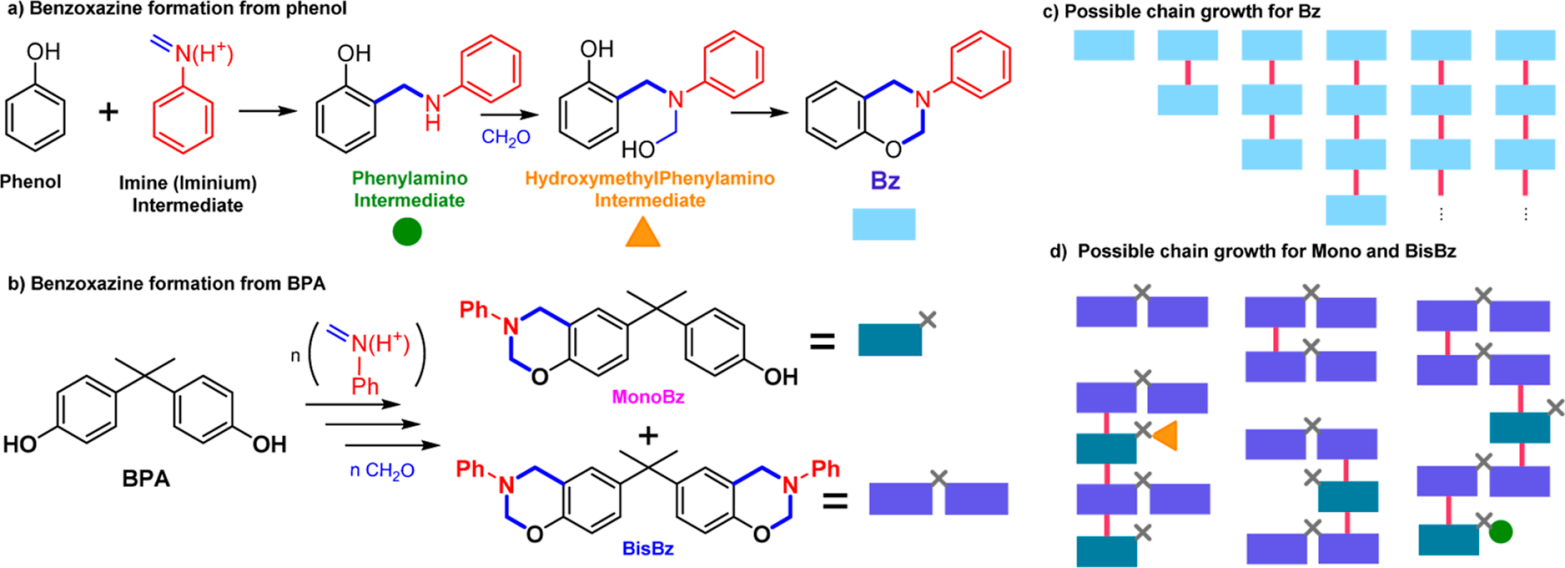
Simplified representation of the (a) benzoxazine synthesis from aniline, phenol, and formaldehyde and (b) the formation of **MonoBz** and **BisBz** from aniline, bisphenol A (**BPA**), and formaldehyde. **BPA** opens different polymerization possibilities, enhancing the molecular diversity as represented in (c) for the **Bz** and (d) for **MonoBz** and **BisBz**.

Previous studies dedicated to the mechanistic understanding of the benzoxazine formation are usually extrapolated for the understanding of bisbenzoxazine reactivity.^10−12^ These studies show that the formation of benzoxazines may be mediated by an iminium intermediate formed by the aniline and formaldehyde reaction that forms a PhenylAmino (**PA**) moiety upon attack of the phenol containing group (Figure 1a). This intermediate reacts with an additional formaldehyde molecule generating a HydroxyMethylPhenilamino intermediate that would lead to the **Bz** product upon cyclization.^1,2^ Further studies also describe that the use of acidic media can facilitate the reaction, even though the last reaction steps are suggested to proceed via a neutral intermediate.^13^

In the context of the bisbenzoxazines, the use of BPA instead of phenol makes it possible that the monobenzoxazine (**MonoBz**) and bisbenzoxazine (**BisBz**) (Figure 1b) formed during the reaction of **BPA**, aniline, and formaldehyde could undergo other lateral reactions. These and other species present in the reaction media could interfere in the polymerization and cure processes, enhancing the molecular diversity and cross-linking degree on the final resin, as represented in Figure 1c,d.

Considering that the formation of bisbenzoxazine does not occur in a synchronous manner at both phenol groups of **BPA**, the formation of bisbenzoxazine (**BisBz**) depends on the formation of the monobenzoxazine (**MonoBz**) or a similar intermediate.

Other interesting characteristic is that the use of **BPA** could allow **MonoBz**-based PA and HydroxyPhenylAmino (**HPA**) intermediates to also take part in the polymerization and side reactions, as represented schematically in Figure 1d.

Therefore, the presence of **BisBz**, **MonoBz**, and their intermediates would greatly increase the possible reactions, making the speciation of the exact isomers present in this reaction media a relevant analytical challenge.

Although tandem mass spectrometry (MS) may be ideal to differentiate isomers in complex matrices because of its high selectivity, low detection limits, and high dynamic ranges,^14−17^ the dissociation patterns and the product ions observed not always allow for the precursor unambiguous identification.^18,19^

For this reason, the combined use of advanced MS techniques as infrared ion spectroscopy (IRIS) and ion mobility spectrometry (IMS) have been recently employed to differentiate isomeric species by MS.^20−26^

Despite the different implementations of IRIS,^27−29^ this technique consists in acquiring the infrared spectra of a mass selected ion by irradiating the species with a tunable infrared radiation and monitoring for ion dissociation. By analyzing the photofragmentation efficiency as a function of the radiation wavenumber, an action IR spectrum of the gaseous phase ion can be acquired and promptly compared to simulated absorption spectra.^30^

IMS may also be implemented in various manners but can be described as a technique to differentiate ions by their interaction with a buffer gas in the presence of static or variable electric fields. During this process, different isomers may experience different interactions with the buffer gas, disturbing their motion. Therefore, different isomers can be differentiated by their distinct collision cross section or mobility parameter.^31,32^

In this context, this work employed these advanced MS techniques for the evaluation of the bisbenzoxazine species formed by aniline, formaldehyde, and **BPA** to characterize the presence of **BisBz**, **MonoBz**, and the intermediates formed during the reaction. Specifically, MS analysis coupled to infrared multiple photon dissociation (IRMPD) spectroscopy and field asymmetric waveform ion mobility spectrometry (FAIMS) were employed. Density functional theory calculations at the B3LYP/6-311+G(d,p) level of theory were carried out to support the experimental IRMPD data.

## Results and Discussion

### Species Observed by Electrospray Ionization–Mass Spectrometry in the Reaction Media

The stoichiometric reaction of **BPA**, formaldehyde, and aniline in the absence of solvent was evaluated by nanospray ionization positive mode MS. The reaction media was sampled after 1 h of reaction at 120 °C and was diluted in acetonitrile to a final concentration of 10^–5^ mol/L of **BisBz**—considering total conversion to this species.

**Figure 2:**
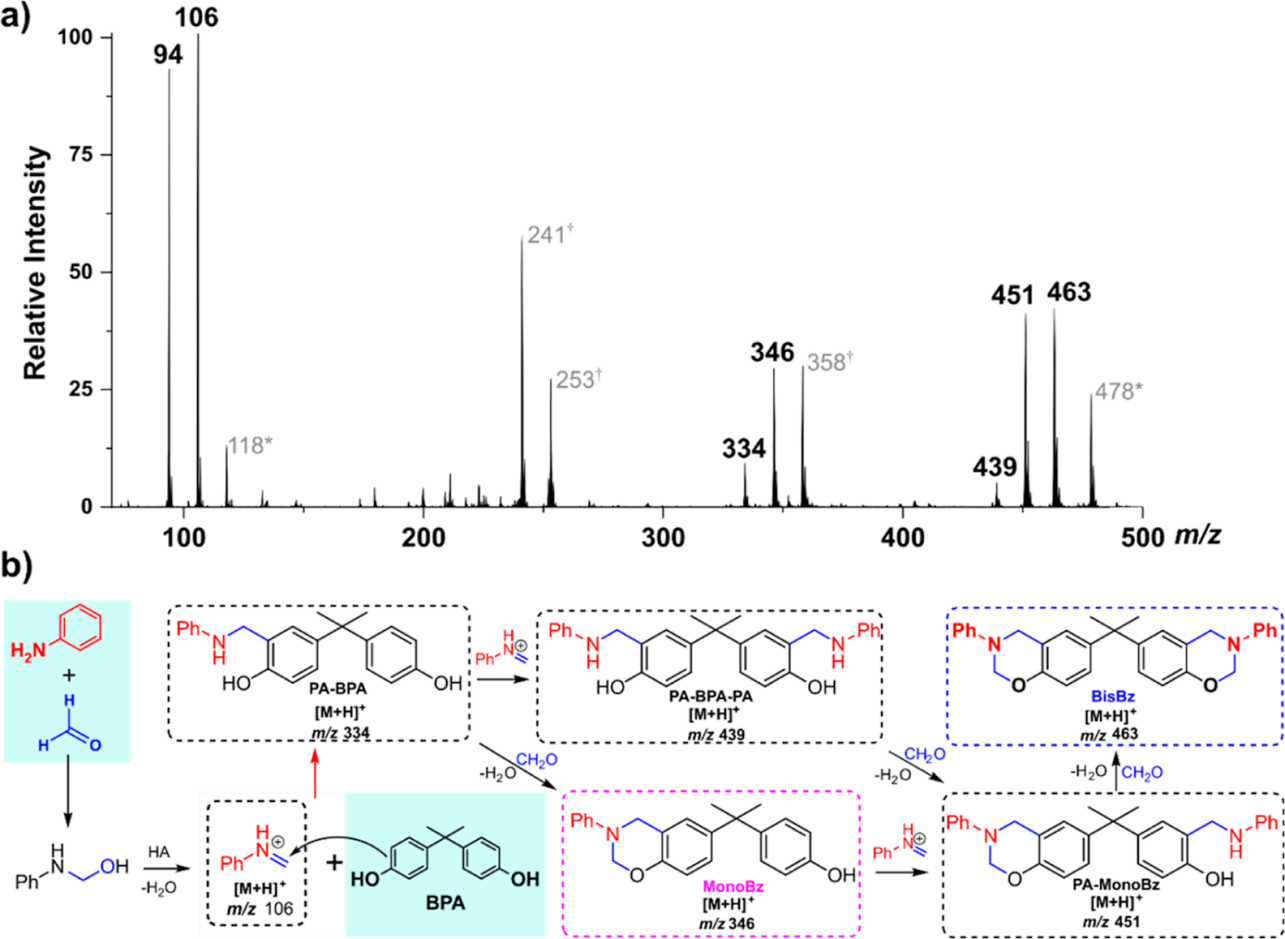
(a) Typical positive mode nanospray ionization MS of a stoichiometric reaction of **BPA**, formaldehyde, and aniline after 1 h of reaction at 120 °C. (b) Possible species observed for the stoichiometric reaction of **BPA**, aniline, and formaldehyde after 1 h at 120 °C, as observed by the positive mode ESI–MS. Isomeric species are not represented and will be discussed throughout the text. Ions marked with † and * are, respectively, in-source fragments and reaction byproducts not relevant for the reaction mechanisms discussed in this work. Their proposed structure can be found in Table S1.

A typical MS spectrum for this reaction medium at the end of the reaction in the positive mode is shown in Figure 2a. This mass spectra show that a series of ions correlated with the formation of **MonoBz** and **BisBz** could be observed, as indicated in Figure 2b and discussed below.

Figure 2a shows signals that were assigned as the aniline-based iminium intermediates with *m*/*z* 106, previously observed for the benzoxazine formation,^2^ and the intermediate with *m*/*z* 334 formed by the attack of the **BPA** to the iminium and the **MonoBz** (*m*/*z* 346). The PA intermediate **PA-MonoBz** formed by the **MonoBz** attack to another iminium species were also observed as the protonated ions with *m*/*z* 451 as well as the **BisBz** species with *m*/*z* 463. An ion with *m*/*z* 439 was assigned as resulting from the attack of the **PA-BPA** intermediate with *m*/*z* 334 to an iminium species forming methylphenylamino intermediate **PA-BPA-PA** at both **BPA** aromatic rings.

It should be considered that there are many possible isomers for the ions depicted in Figure 2, and this is especially relevant as the isomeric species for the benzoxazine synthesis were previously shown to influence the reaction outcome.^33^ For this reason, the detected species pointed out previously were submitted to ion spectroscopy and ion mobility studies so their nature could be unambiguously determined.

### Identification of Reaction Intermediates

**Figure 3:**
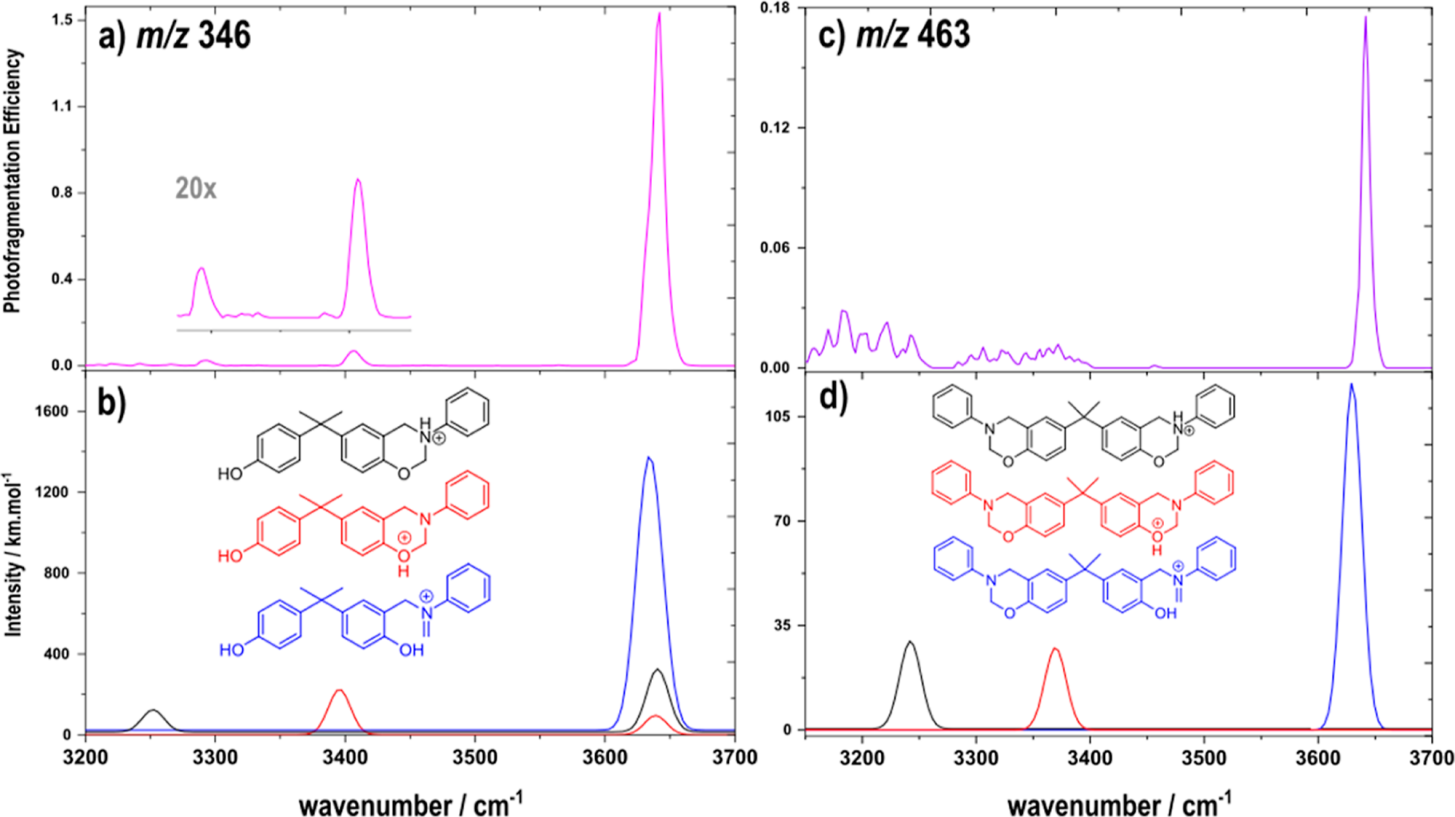
(a) IRMPD spectrum of the species with *m*/*z* 346, (b) calculated spectra of the **N-MonoBz** (black), **O-MonoBz** (red), and **ROP-MonoBz** (blue) isomers, (c) IRMPD spectrum of the species with *m*/*z* 463, and (d) calculated spectra of the **N-BisBz** (black), **O-BisBz** (red), and **ROP-BisBz** (blue). The simulated absorption spectra were calculated at the B3LYP/6-311+G(d,p) level of theory using 0.95 as a scale factor.

IRMPD spectroscopy was used to evaluate the nature of the intermediates observed in this reaction media, as shown in Figure 3.

In the experimental IRMPD spectrum of protonated **MonoBz** with *m*/*z* 346 (Figure 3a), three different bands are observed. The most intense at 3641 cm^–1^ can be assigned to the phenolic OH stretch of the **ROP-MonoBz** isomer that was calculated at 3635 cm^–1^ (Figure 3b—blue). This **ROP-MonoBz** species is analogous to the ring-opening polymerization (ROP) intermediate described in the literature as the initial species in the polymerization of benzoxazines.^13,34^

The lower intensity bands observed at 3406 and 3292 cm^–1^ on the experimental IRMPD spectrum were assigned to the **MonoBz** species protonated at the benzoxazine oxygen atom (**O-MonoBz**) and N protomer (**N-MonoBz**) as the theoretical absorption spectra for these species show the protonated O–H^+^ and N–H^+^ stretches at 3396 and 3253 cm^–1^, respectively (Figure 3b—red and purple).

Figure 3c shows the IRMPD spectrum of the protonated **BisBz** with *m*/*z* 463, which presents one intense band in the phenolic OH stretch region at 3642 cm^–1^ assigned to the **ROP-BisBz** species (Figure 3d blue) and two other minor bands at 3370 and 3208 cm^–1^. Similar to the **MonoBz** species, **BisBz** can also be presented as the N and O protomers (**N-BisBz** and **O-BisBz**, respectively) that show an N–H stretch calculated at 3284 cm^–1^ and an OH^+^ stretch at 3399 cm^–1^, explaining the minor bands observed at 3250 and 3400 cm^–1^ regions. It should be noted that the low calculated intensity of these bands (<50 km·mol^–1^) is consistent with the higher noise observed at this region as the a lower absorption of the laser radiation causes a reduction of the photodissociation efficiency.^35^

**Figure 4:**
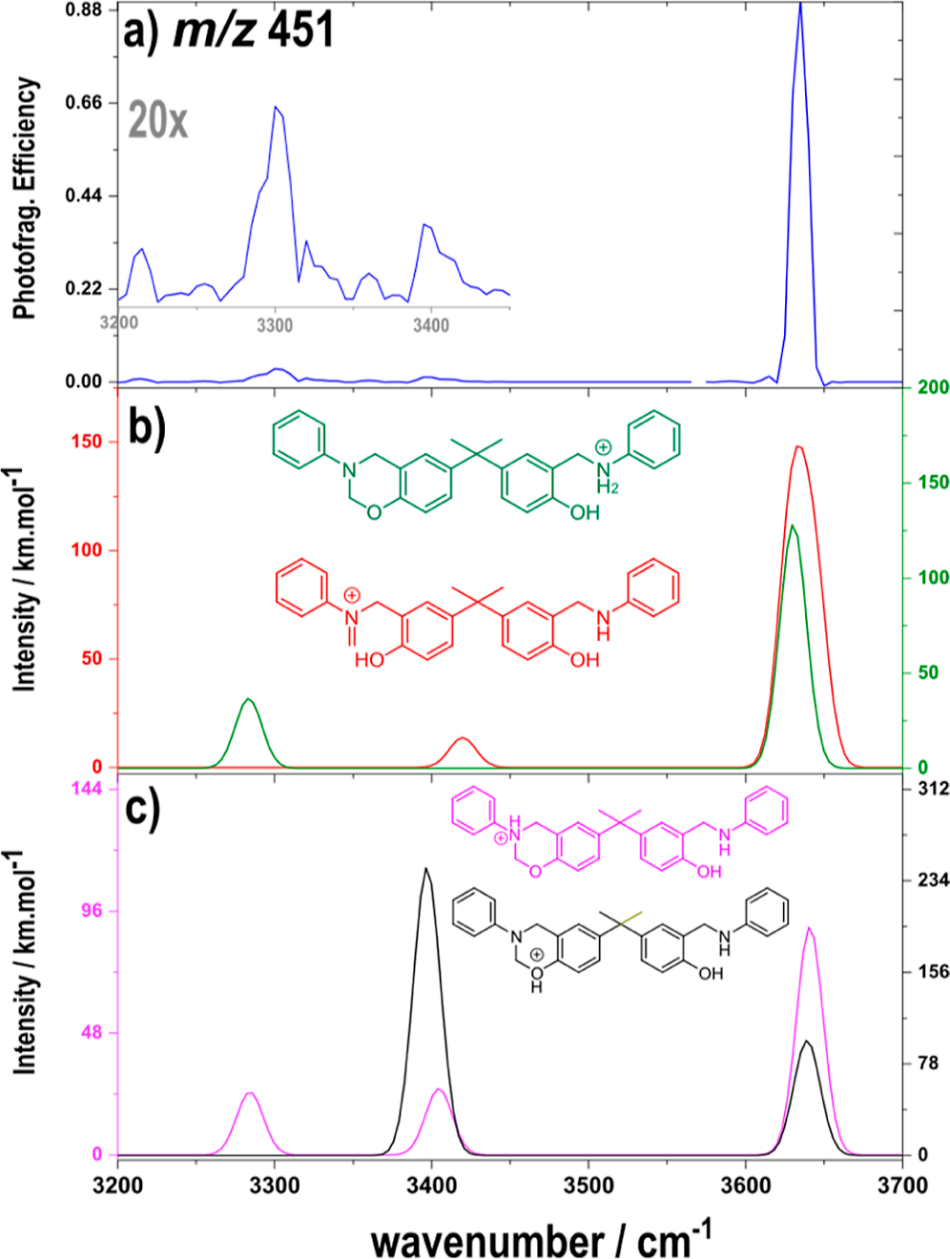
(a) IRMPD spectrum of the species with *m*/*z* 451 (blue), (b) calculated spectra of the **NH-PA-MonoBz** (green) and **ROP-PA-MonoBz** species (red), and (c) calculated spectrum of the **N-PA-MonoBz** (pink) and **O-PA-MonoBz** (black) species at the B3LYP/6 311+G(d,p) level of theory using 0.95 as a scale factor.

Figure 4a shows the IRMPD spectrum for the intermediate with *m*/*z* 451, assigned as the PA intermediated **PA-MonoBz**.

This spectrum shows mainly one intense absorption at 3635 cm^–1^, in accordance with the phenolic stretching (O–H) observed for the other IRMPD reported in this work. Similar to **MonoBz**, this **PA-MonoBz** can be presented as the ROP intermediate **ROP-PA-MonoBz** and the PA protonated protomer **NH-PA-MonoBz** (Figure 4b), besides the N and O protomers **O-PA-MonoBz** and **N-PA-MonoBz** (Figure 4c). The OH stretch obtained from the calculated absorption spectra of **O-PA-MonoBz** and **N-PA-MonoBz** protomers were 3634, 3631 cm^–1^ and 3640 and 3641 cm^–1^, respectively (Figure 4c). The **ROP-PA-MonoBz** show calculated OH stretches at 3642 and 3629 cm^–1^, and the PA protonated protomer **N-PA-MonoBz** (Figure 4b) that shows one OH stretch at 3629 cm^–1^.

Some less intense bands in the 3000–3450 cm^–1^ range can also be observed. The band at 3400 cm^–1^ could be correlated to the theoretical spectra of **ROP-PA-MonoBz**, **N-PA-MonoBz**, or **O-PA-MonoBz** as all these species show signals in this same region. Similarly, the other experimental band at 3301 cm^–1^ can be related to both **N-PA-MonoBz** and **NH-PA-MonoBz**.

It should be noted that the protonated **PA-BPA** and **PA-BPA-PA** intermediates were also subjected to IRMPD spectroscopy, confirming their nature by the presence of absorption bands assigned to the NH and OH stretches (Figures S1 and S2).

**Figure 5:**
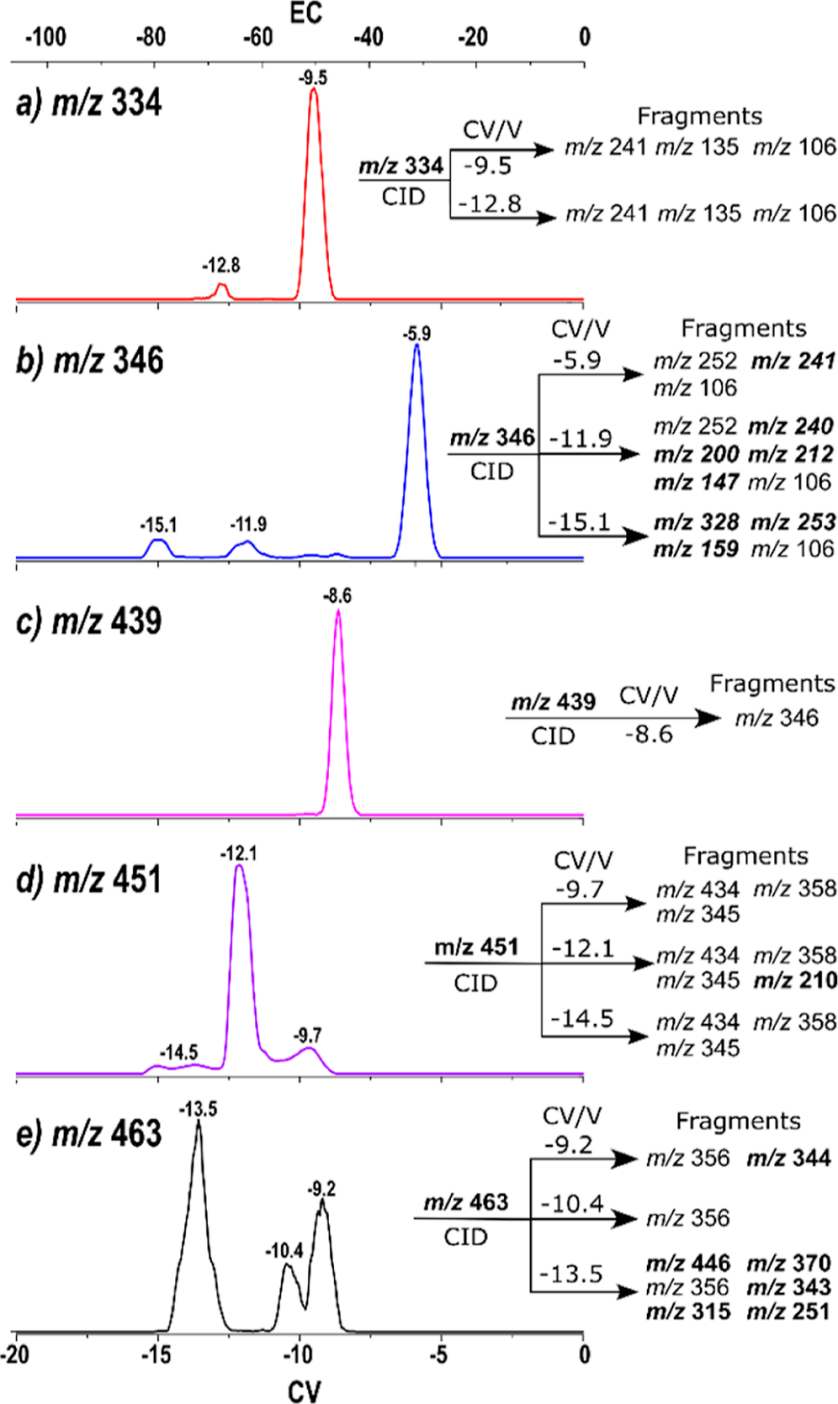
FAIMS spectra for the ion (a) *m*/*z* 334 (**PA-BPA**); (b) *m*/*z* 346 (**MonoBz**); (c) *m*/*z* 439 (**PA-BPA-PA**); (d) *m*/*z* 451 (**PA-MonoBz**); and (e) *m*/*z* 463 (**BisBz**). Each spectra represents the fragments observed for each population (Figures S5–S7). Highlighted species indicate population-specific fragments.

The intermediates and **BisBz** species observed were also subjected to ion mobility analysis (FAIMS), as shown in Figure 5.

Figure 5a shows the FAIMS spectrum for PA intermediate **PA-BPA** with *m*/*z* 334. This ion mobility spectrum shows one major population at a compensation voltage (CV) of −9.5 V and a minor one at CV −12.8 V. These two isomeric populations can be explained by the reactivity of the phenolic compound, **BPA**, which may allow the formation of the *meta*-substituted PA-substituted BPA (*m*-**PA-BPA**). As expected, the *ortho*-substituted **PA-BPA** should be the main population present. It is worth mentioning that the *ortho*-substituted **BPA** is identified solely as **PA-BPA** in this text instead of *o*-**PA-BPA** for simplicity. For both populations, the MS/MS spectrum obtained by collision-induced dissociation (CID) shows the same product ions (*m*/*z* 241, 135, and 106) and cannot be used to differentiate these populations (Figure S3).

Conversely, the intermediate **PA-BPA-PA** with *m*/*z* 439 (Figure 5c) presents just one population at CV −8.6 V. This is because the formation of this intermediate as the *ortho* substitution in both aromatic rings of BPA is statistically favored from the major **PA-BPA** present. The fragmentation of this species produces an ion with *m*/*z* 346 (Figure S4).

The FAIMS spectrum of the **MonoBz** intermediate with *m*/*z* 346 (Figure 4b) shows three main populations at CVs −15.1, −11.9, and −5.9 V. These three populations can be correlated to the three IRMPD bands observed for this species (Figure 3a).

To distinguish these three populations, MS/MS spectra of the selected populations were acquired (Figure S5). The relevant fragments for each population are shown in Figure 5, where population-specific fragments are highlighted in bold.

The fragmentation pattern for the population at −15.1 V is consistent with **ROP-MonoBz**, as suggested by the formation of the fragment with *m*/*z* 328, associated with a loss of a water molecule from the phenolic OH group. The fragments at *m*/*z* 253 is characteristic of alkyne fragments, originating from condensed cyclic compounds.^36^

Although specific fragments with *m*/*z* 240, 200, 212, and 147 were observed for the population at −11.9 V, and with *m*/*z* 241 for the population at −5.9 V, no clear fragmentation mechanism could be proposed to differentiate these populations. It should also be considered that depending on the potential landscape for dissociation, there could be an interconversion between these isomers that may prevent their differentiation by CID.^37^

Figure 5d shows the FAIMS spectrum of protonated **PA-MonoBz** with *m*/*z* 451. It is possible to notice one major population at −12.1 V, a second population at −9.7 V, and two other unresolved minor features at −14.5 V. The complexity of the IRMPD spectra for this species suggested that all possible isomers proposed (different protomers and the ROP intermediate) could be present, as supported by the number of populations observed in this mobility spectrum.

Population-specific CID results for the protonated **PA-MonoBz** show a fragment with *m*/*z* 210 for the population at −12.1 V (Figure S6), suggesting the **PA-MonoBz** is protonated at the nonoxazinic nitrogen.

The protonated **BisBz** species, *m*/*z* 463, showed a similar result to the **MonoBz**, presenting three populations that support the IRMPD data. The population-specific CID mechanism for this ion shows the formation of an ion with *m*/*z* 356 for the three populations besides some population-specific fragments (Figure S7). From those fragments, the one with *m*/*z* 446 suggests that the population at CV −13.5 V could be assigned to the **ROP-BisBz** as this species would have a free OH group to justify loss of 17 Da.

In summary, although not all the fragments could be assigned, the CID for specific populations allowed differentiation of the OH containing isomers associated with the presence of the ROP intermediate and suggests their presence in the bisbenzoxazine reaction media.

### Overall Reaction Mechanism

**Figure 6:**
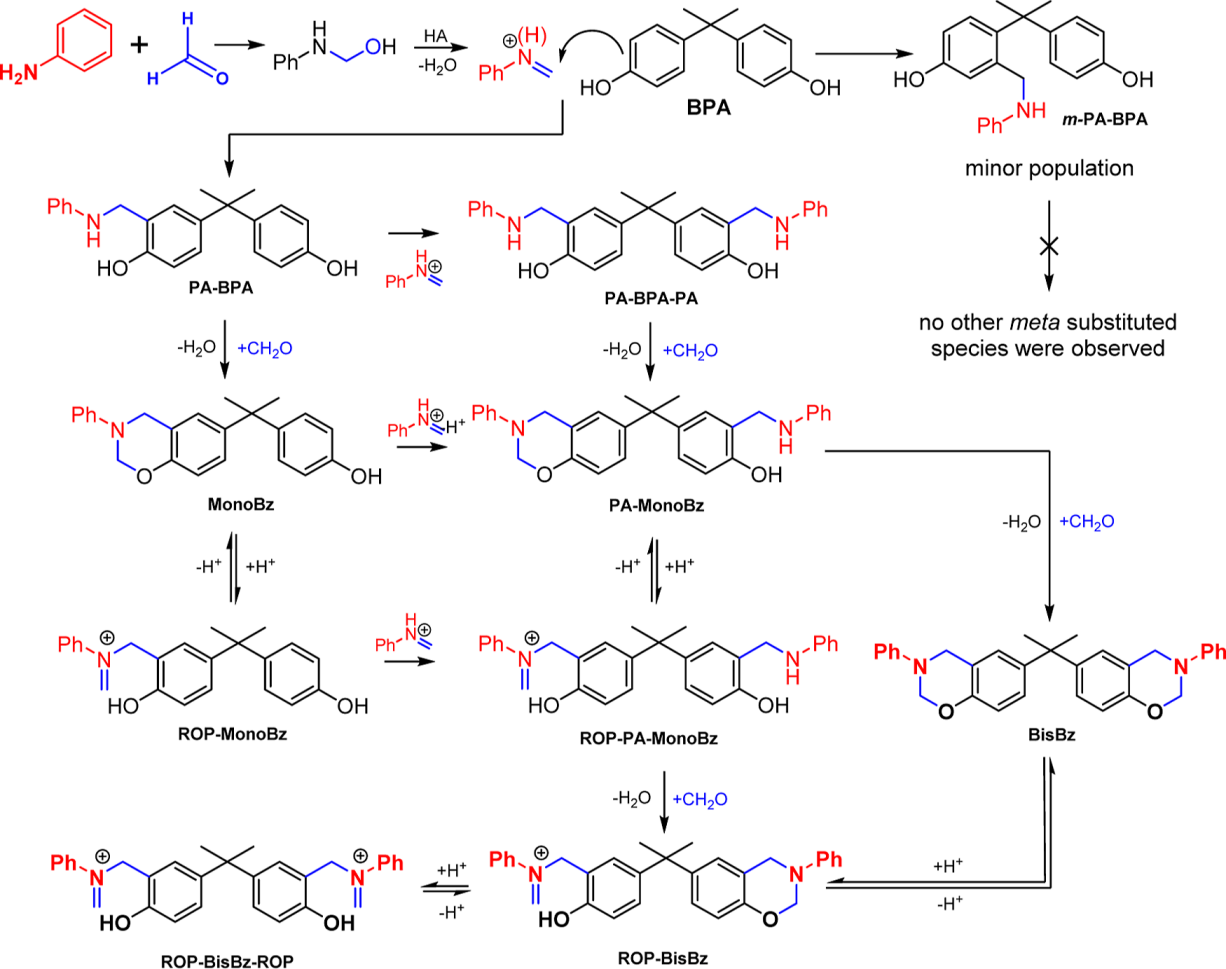
Representation of the most relevant species for the **BisBz** monomer formation as discussed in the text. All *o*-**PA** intermediates represented are identified by **PA** only for clarity.

Considering the results presented in this work, Figure 6 shows a detailed picture of the most relevant species present in the reaction media.

It is relevant to point out that this general mechanism suggests that the formation and extension of **MonoBz**, **BisBz**, and the other intermediates present in the reaction media are dependent on the competition of two reaction pathways: (i) the attack of an aromatic center to the imine or iminium intermediate and (ii) the attack of the aminoaromatic species to the formaldehyde, generating the benzoxazine ring after dehydration. Considering the relative intensities typically observed for the **PA-BPA-PA** are much lower than the **MonoBz** species (Figure 2a) and supposing that the relative ionization and detection efficiency of this two species are similar,^38,39^ it is possible to suggest that the aromatic attack to the formaldehyde is the most favorable of these two reactions as the opposite would lead to minor quantities of **MonoBz** being formed during the reaction. No HydroxyPhenylAmino (**HPA**) intermediates were observed during this study, suggesting that they fast proceed to the **PA** intermediates.

Figure 6 shows that the bisbenzoxazines synthesis produces many species that may influence the polymerization and cure processes. It is also interesting to see that there is a pathway that goes from the initial reactants to the ROP intermediates **ROP-BisBz** and **ROP-BisBz-ROP** (Figure 6, last line) that are formed by the **ROP-PA-MonoBz** intermediate and not by the **BisBz** monomer. This consideration shows that the knowledge of the species that are effective in the reaction media allows for better planning of the reaction procedures and understanding of the reactions involved in the benzoxazines and PBz production.

## Conclusions

This study has detected and identified the isomeric intermediates in the model reaction of **BPA** and formaldehyde and aniline to form bisbenzoxazine. The species observed in positive mode nanospray MS were further investigated by IRMPD spectroscopy and FAIMS. The competition between the: (i) **BPA** aromatic attack to the imine or iminium intermediate and (ii) the attack of the aminoaromatic species to the formaldehyde generate concurrent intermediates that can be prone to further side reactions.

The **PA** intermediates were shown to be formed almost exclusively at the ortho position, while the oxazine containing intermediates (**MonoBz**, **BisBz**, and **PA-MonoBz**) are suggested to also be present as the iminium **ROP** intermediate and as N- and O-protomers. No hydroxiphenylamino intermediates were observed in this work despite their presence in previous studies on the benzoxazine formation.^2^

The nature of the isomeric species present in the reaction media was confirmed by IRMPD spectroscopy and population-specific CID data. Although isomer specific CID data show specific fragments for the isomers, no general mechanism could be proposed to rationalize these results except for the OH containing isomers associated with the ROP intermediate.

These results were considered to propose a comprehensive description of the species present in the reaction media of the **BisBz** formation, pointing out the intermediates and species prone to polymerization that would take place in chain termination and cross-linking steps observed during the polymerization of these species.

## Experimental Section

### Reaction Procedure and Sample Preparation

The bisbenzoxazine (2,2-bis(3,4-dihydro-3-phenyl-2*H*-1,3-benzoxazine)propane) synthesis was carried out by adding 3.1 mmol 2,2-Bis(4-hydroxyphenyl)propane (bisphenol – TCI, ≥90.0%), aniline 6.2 mmol (Sigma, ≥99.5%), and 12.4 mmol of formaldehyde (Sigma, 37%) to a borosilicate glass flask. The reaction media was stirred for 1.0 h at 120 °C by a magnetic stirer.^40^ When the reaction was over, 10 mL of ethyl acetate (ACS grade, Synth) was added to solubilize the reaction media. This solution was then sampled and diluted in acetonitrile (high-performance liquid chromatography grade, Sigma-Adrich) so that a final concentration of **BisBz** of 10^–5^ mol/L was obtained, considering full conversion to products. 1.0 μL of formic acid was added to this solution before the MS analysis. The acid addition immediately before analysis is shown in previous studies to enhance ion intensities without disturbing the reaction media.^13^

### MS Analysis

The full scan MS, CID, and IMS data were acquired in the positive mode via a nanoelectron spray ionization source using a modified Thermo LTQ XL linear ion trap mass spectrometer. The original instrument inlet and source assembly were substituted with an electrodynamic ion funnel (HeartLand MS). The internal high voltage source of the instrument were used to apply 3.0 kV via a platinum wire to the solution inside a borosilicate glass capillary (BF165-120-10—Sutter Instrument Company) with a 2 μm ID tip prepared by a Sutter Instrument Company P-2000 micropipette puller (programing available at Supporting Information).^41^

The CID experiments were carried out using He as the collision gas (Air Products, 99.999%), and the normalized collision energy values employed varied from up to 25%.

For the IMS analysis, a planar field asymmetric waveform ion mobility spectrometer (FAIMS—Heartland MS) with a gap width of 1.89 mm and operating at ambient pressure and temperature was coupled to the electrodynamic ion funnel interface. In FAIMS the ions are separated by their differential ion mobility in the high and low electric field regimen promoted by a bisinusoidal waveform (1 MHz frequency, 2:1 harmonic ratio, dispersion voltage = 4.5 kV). This asymmetric field allows the ions to interact with the buffer gas, generating spatial separation dependent on the ion’s mobility parameters. These ions can be refocused on the mass spectrometer inlet by a CV that allows their differentiation, generating a plot of ion intensity as a function of the CV.

In this work, the CV was scanned at 4 V·min^–1^ from −20 to 0 V in relation to a 200 V bias voltage to guarantee that the FAIMS device was at higher potential than the ion funnel entrance. A Matsusada (HJPM-1R15) power supply was used to apply 1.4 kV to the FAIMS cell curtain plate. A 3.0 L/min flow of N_2_ used as the drying and buffer gas were supplied from evaporation from liquid nitrogen (White Martins) and controlled by a digital flow meter (Sensirion SFC5500) operating at a 3.0 psi.^42,43^

IRMPD spectra were recorded in the 2800–3800 cm^–1^ range by coupling the output beam of an optical parametric oscillator/amplifier (OPO/OPA) (LaserVision, ∼12 mJ/pulse, 3.7 cm^–1^ resolution) pumped by a 10 Hz Nd:YAG laser (Continuum Surelite II, 570 mJ/pulse) to a modified Bruker AmaZon SL 3D ion trap mass spectrometer, as described in details elsewhere.^44,45^ The photofragmentation efficiency at a given wavenumber ν, Eff_ν_, was calculated according to the equation Eff_ν_ = −ln((*P*_ν_)/(*P*_ν_ + Σ*F*_jν_)), where *P*_ν_ represents the parent ion intensity at a given wavelength; and ν and *F*_jν_ represent the jth fragment ion intensities at the same wavenumber ν. The laser beam power was measured by a Coherent J-10MB-LE and a SpectraPhysics 407A power meter before and after the ion trap, respectively. Irradiation times were determined by monitoring the dissociation of the most intense absorption, so the fragment ion was approximately 10% of the parent intensity and varied from 1000 to 2000 ms (10 to 20 pulses) in this study.

## Computational Methods

Quantum mechanical calculations were carried out using Gaussian 16 (Revision C.01)^46^ employing the hybrid B3LYP functional^47^ for optimizations and frequency calculations at the 6-311+G(d,p) basis set at 25 °C under vacuum. The suitability of this level of theory was discussed in literature.^45^ Vibrational analysis showed the absence of imaginary frequencies in the species reported in this work and was compared to the experimental IRMPD spectra by using a scale factor of 0.95.^48^

## Supporting Information Available

Details on the preparation of nanospray capillaries, IRMPD spectra of ions with *m*/*z* 334 and 439, MS^2^ spectra of all species discussed in this work, along with fragment identification, assignment of byproducts present, and atomic coordinates with 3D renderings of all geometrical structures supporting IRMPD assignments (PDF)
