# Supplementary material for: Isomeric Speciation of Bisbenzoxazine Intermediates by Ion Spectroscopy and Ion Mobility Mass Spectrometry
Source: ACS Omega. 2024 Sep 18;9(39):40932–40. doi: 10.1021/acsomega.4c06205 (PMC11447905; doi:10.1021/acsomega.4c06205)
Supplement: Supplementary file 1 — ao4c06205_si_001.pdf [file ao4c06205_si_001.pdf]

Isomeric Speciation of Bisbenzoxazine Intermediates by Ion Spectroscopy and Ion Mobility Mass Spectrometry

Francisco W. M. Ribeiro; Danilo Silva-Oliveira; Gustavo Cervi; Eduardo D. Koyanagui and Thiago C. Correra*

Department of Fundamental Chemistry, Institute of Chemistry, University of São Paulo Av. Prof. Lineu Prestes, 748, Cidade Universitária, São Paulo, São Paulo, 05508-000, Brazil.

Correspondence

Thiago C. Correra, Department of Fundamental Chemistry, Institute of Chemistry, University of São Paulo Av. Prof. Lineu Prestes, 748, Cidade Universitária, São Paulo, São Paulo, 05508-000, Brazil. E-mail: tcorrera@iq.usp.br

Details on the preparation of nanospray capillaries 2

Figure S1. a) IRMPD spectrum of compound with *m/z* 334 (PA-BPA) and b) theoretical absorption spectra for the putative structure calculated at the B3LYP/6-311+G(d,p) level of theory using 0.95 as scale factor. 3

Figure S2. a) IRMPD of compound with *m/z* 439 (PA-BPA-PA) and b) theoretical absorption spectra for the putative structure calculated at the B3LYP/6-311+G(d,p) level of theory using 0.95 as scale factor. 4

Figure S3. MS^2 spectra of specific populations (different CV values) of the ion with *m/z* 334 (PA-BPA). 5

Figure S4. MS^2 spectra of specific populations at -8.6 V(CV value) of the ion with *m/z* 439. 6

Figure S5. MS^2 spectra of the ion with *m/z* 349 at specific populations selected by their CV values: a) -5.9 V, b) -11.9 V and c) -15.1 V....................7

Figure S6. MS^2 spectra of the ion with *m/z* 451 at specific populations selected by their CV values: a) -9.7 V, b) -12.1 V and c) -14.5 V. 8

Figure S7. MS^2 spectra of the ion with *m/z* 463 at specific populations selected by their CV values: a) -9.2 V, -10.4 V and -13.5 V....................9

Table S1. Putative fragment identification and assignment of byproducts present in Figure 1 representing the species detected by ESI(+)-MS during the BisBz synthesis. 10

Atomic coordinates with 3D renderings of all geometrical structures supporting IRMPD assignments 11

Details on the preparation of nanospray capillaries

Program for BF165-120-10 capillary:

First step: Heat, pull 0, vel 11, time 0;

Second step: Heat-50, pull 0, vel 11, time 0;

Third step: Heat+100, pull 0, vel 3, time 231;

Fourth step: Heat-50, pull 0, vel 170, time 231.

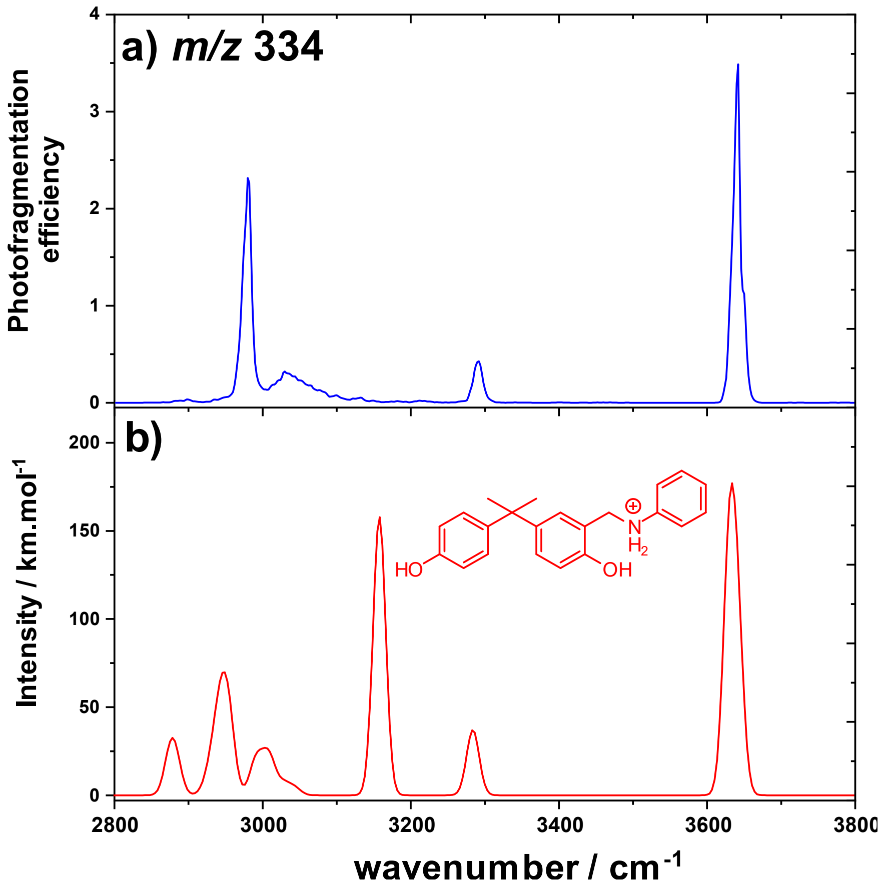

Figure S1. a) IRMPD spectrum of compound with *m/z* 334 (**PA-BPA**) and b) theoretical absorption spectra for the putative structure calculated at the B3LYP/6-311+G(d,p) level of theory using 0.95 as scale factor.

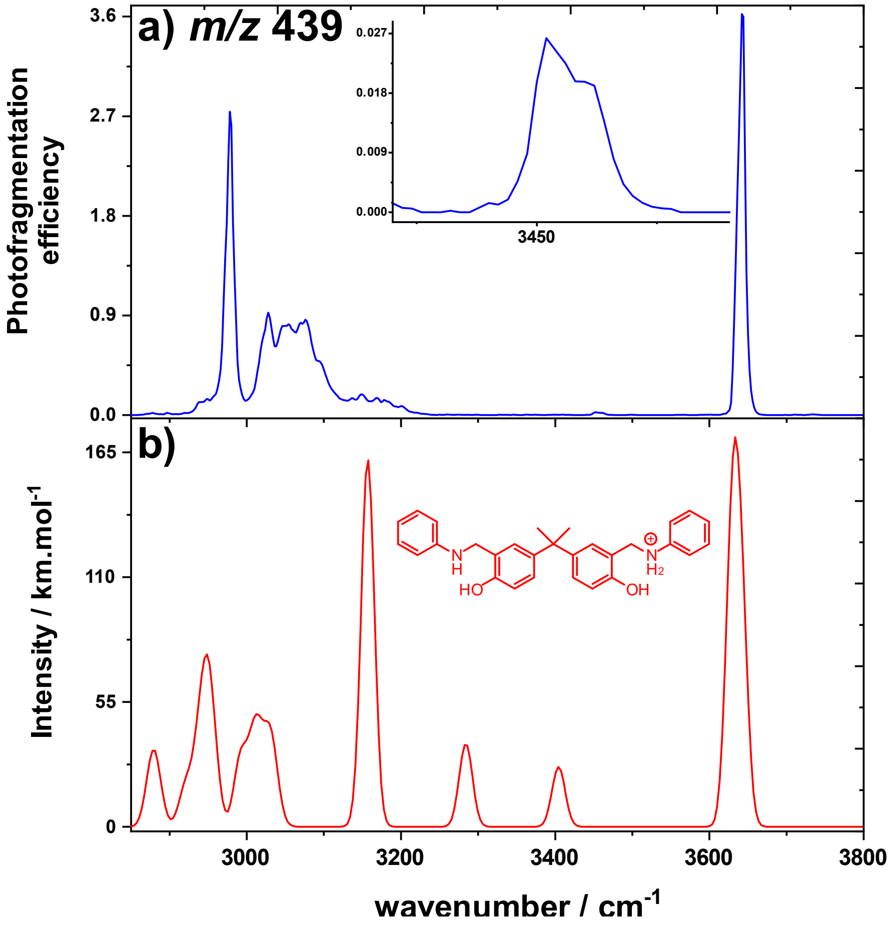

Figure S2. a) IRMPD of compound with *m/z* 439 (**PA-BPA-PA**) and b) theoretical absorption spectra for the putative structure calculated at the B3LYP/6-311+G(d,p) level of theory using 0.95 as scale factor.

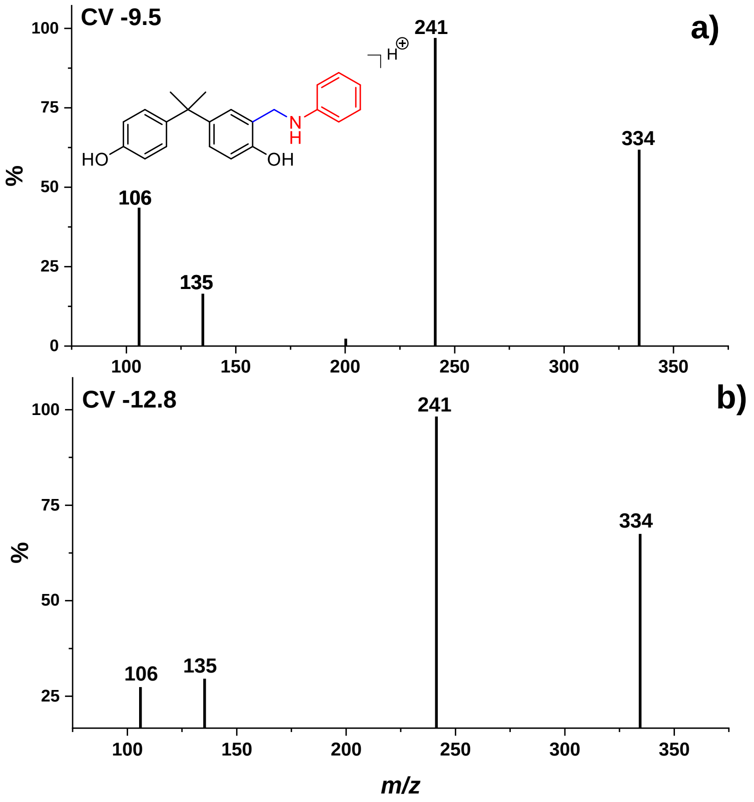

Figure S3. MS^2 spectra of specific populations (different CV values) of the ion with *m/z* 334 (**PA-BPA**).

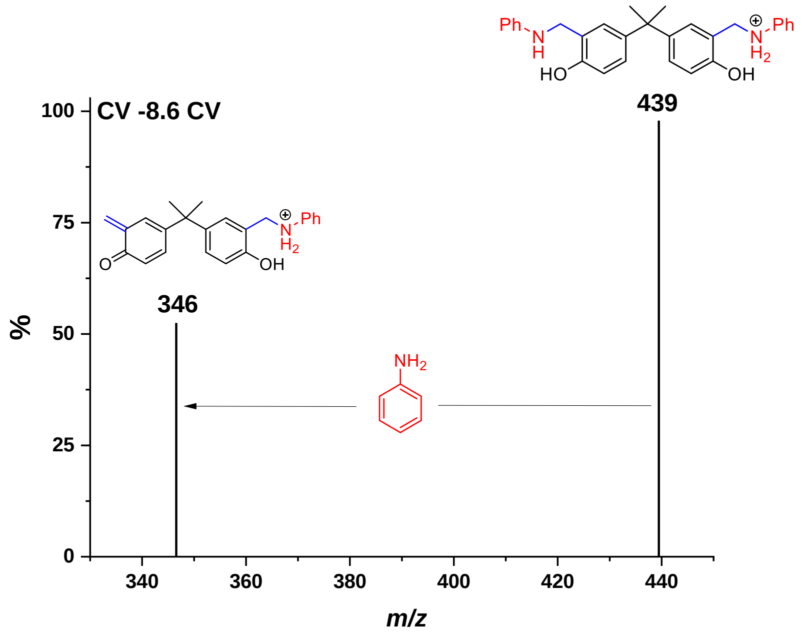

Figure S4. MS^2 spectra of specific populations at -8.6 V(CV value) of the ion with *m/z* 439.

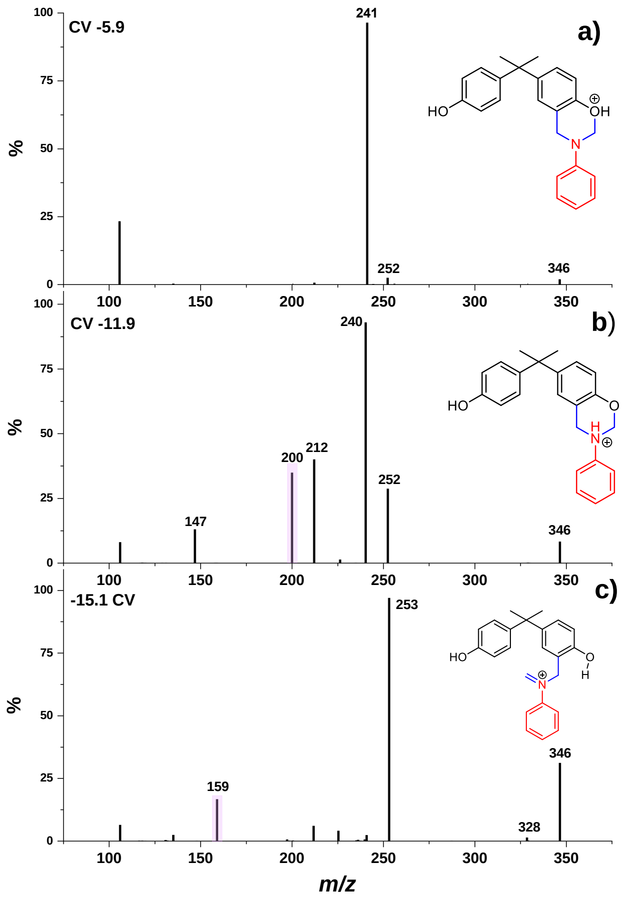

Figure S5. MS^2 spectra of the ion with *m/z* 349 at specific populations selected by their CV values: a) -5.9 V, b) -11.9 V and c) -15.1 V.

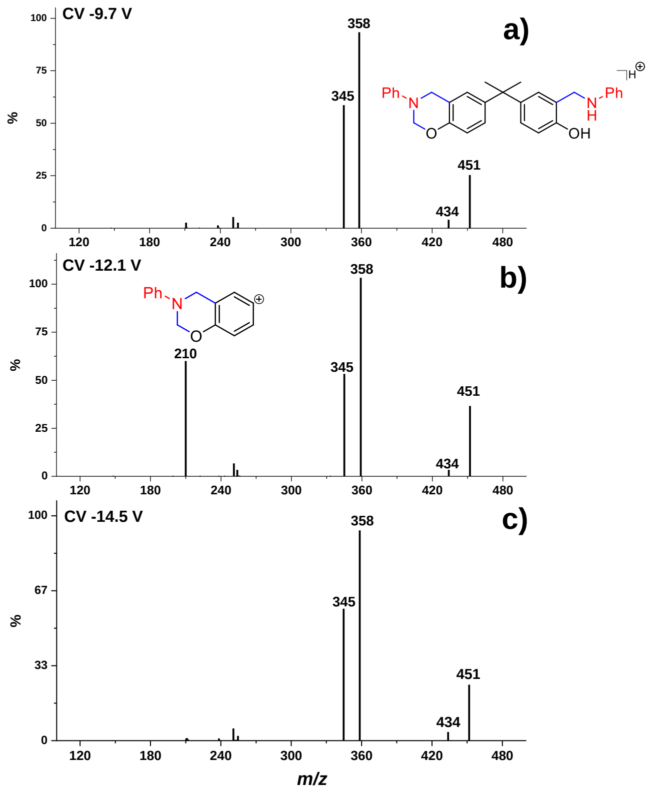

Figure S6. MS^2 spectra of the ion with *m/z* 451 at specific populations selected by their CV values: a) -9.7 V, b) -12.1 V and c) -14.5 V.

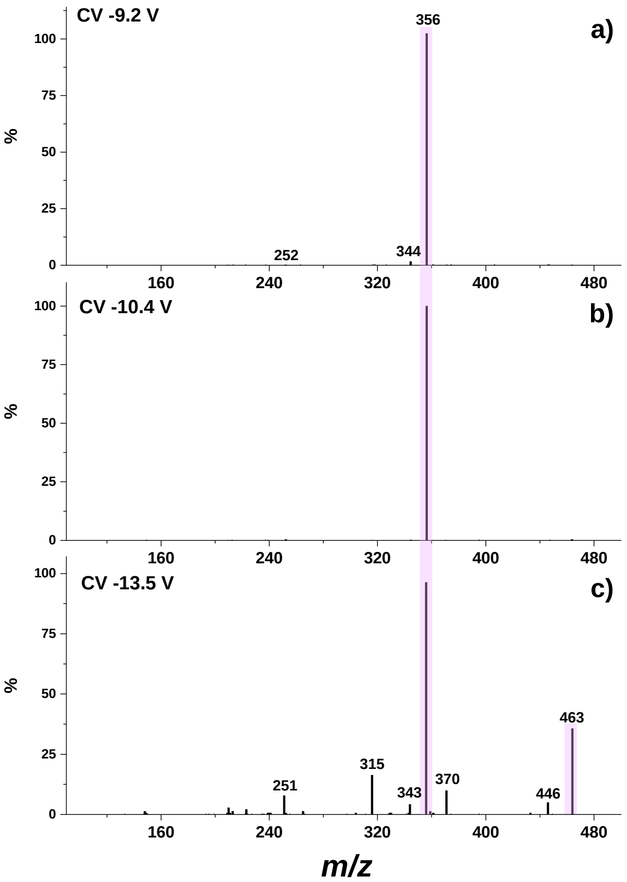

Figure S7. MS^2 spectra of the ion with *m/z* 463 at specific populations selected by their CV values: a) -9.2 V, -10.4 V and -13.5 V.

Table S1. Putative fragment identification and assignment of byproducts present in Figure 1 representing the species detected by ESI(+)-MS during the **BisBz** synthesis.

m/z	Structure
118	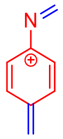
241	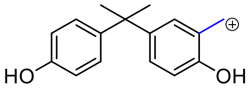
253	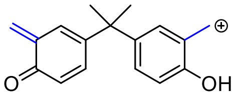
358	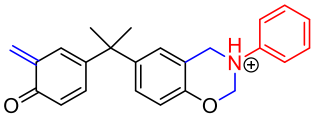
478	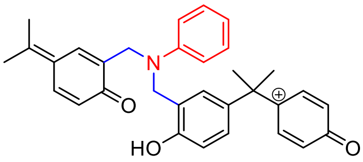

Atomic coordinates with 3D renderings of all geometrical structures supporting IRMPD assignments

Coordinates				molecule
6	-0.897474000	-1.599127000	-1.010577000	
6	0.397271000	-1.941487000	-1.379663000	
6	-1.129221000	-0.846659000	0.143599000	
1	0.576835000	-2.517996000	-2.281705000	
6	1.462808000	-1.534677000	-0.584991000	
6	-0.035383000	-0.440522000	0.919908000	
1	2.468566000	-1.798662000	-0.889515000	
1	-0.231510000	0.138244000	1.814480000	
6	1.276155000	-0.778487000	0.582099000	
6	2.491412000	-0.415624000	1.458441000	
6	3.623900000	0.131192000	0.566953000	
6	4.913060000	-0.413013000	0.548513000	
6	3.388626000	1.246804000	-0.247153000	
1	5.154610000	-1.273577000	1.158666000	
1	2.403077000	1.700288000	-0.270764000	
6	5.924701000	0.126953000	-0.241294000	
6	4.387350000	1.799769000	-1.041200000	
1	6.918761000	-0.303255000	-0.243282000	
1	4.173087000	2.666571000	-1.659403000	
6	5.667202000	1.238979000	-1.040021000	
6	2.148132000	0.654338000	2.521137000	
1	3.051991000	0.911631000	3.075957000	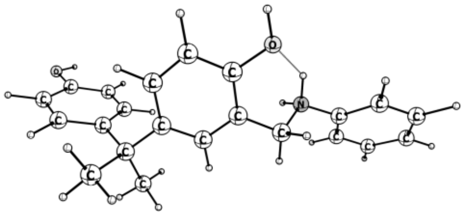
1	1.767020000	1.574329000	2.071699000	
1	1.414258000	0.287428000	3.244843000	
6	2.903480000	-1.703809000	2.211805000	
1	3.200479000	-2.502905000	1.529233000	
1	3.733268000	-1.507998000	2.894762000	
1	2.062989000	-2.069303000	2.806591000	
8	-2.015334000	-1.943507000	-1.755207000	
1	-1.776254000	-2.521805000	-2.489192000	**PA-BPA**
8	6.693985000	1.726615000	-1.793279000	
1	6.412671000	2.509133000	-2.279184000	
6	-2.536506000	-0.531410000	0.554136000	
1	-3.138447000	-1.424580000	0.728946000	
1	-2.579814000	0.103326000	1.437676000	
7	-3.274178000	0.219542000	-0.568256000	
1	-3.197839000	-0.388895000	-1.396799000	
6	-4.685759000	0.555410000	-0.290759000	
6	-4.984012000	1.783988000	0.285746000	
6	-5.667683000	-0.381178000	-0.590610000	
1	-4.202385000	2.503308000	0.508286000	
1	-5.411972000	-1.332744000	-1.044297000	
6	-6.315378000	2.082484000	0.568279000	
6	-6.994650000	-0.069710000	-0.301363000	
1	-6.566899000	3.037980000	1.011534000	
1	-7.774032000	-0.785065000	-0.532883000	
6	-7.316510000	1.157272000	0.276868000	
1	-8.350161000	1.394881000	0.496616000	
1	-2.747387000	1.069095000	-0.785713000	

6	-2.950257000	-0.383569000	-0.906483000
6	-1.670699000	-0.902835000	-0.927442000
6	-3.354444000	0.746426000	-0.224489000
1	-1.424729000	-1.784314000	-1.506975000
6	-0.714851000	-0.226002000	-0.182547000
6	-2.352245000	1.400825000	0.505841000
1	0.305321000	-0.588717000	-0.186653000
1	-2.628620000	2.295840000	1.049481000
6	-1.035854000	0.933546000	0.549443000
6	0.057454000	1.605276000	1.402349000
6	1.337569000	1.755973000	0.557300000
6	2.571366000	1.214098000	0.935306000
6	1.297180000	2.492329000	-0.634135000
1	2.663937000	0.640420000	1.848377000
1	0.363105000	2.931112000	-0.969694000
6	3.716412000	1.397224000	0.165243000
6	2.431341000	2.684872000	-1.414938000
1	2.367940000	3.264167000	-2.331139000
6	3.652800000	2.136076000	-1.014885000
6	-0.366669000	3.002410000	1.910800000
1	0.463640000	3.447204000	2.461511000
1	-0.617527000	3.682027000	1.093506000
1	-1.219037000	2.946216000	2.594060000
6	0.267945000	0.701449000	2.642554000
1	0.604351000	-0.301878000	2.372966000
1	1.005171000	1.141369000	3.317433000
1	-0.669961000	0.603797000	3.194541000
8	-3.986910000	-1.093457000	-1.717823000
8	4.803768000	2.283313000	-1.727973000
6	-4.780345000	1.262593000	-0.325030000
1	-5.185638000	1.476272000	0.663795000
1	-4.778589000	2.209157000	-0.877348000
7	-5.681458000	0.324667000	-1.014190000
6	-6.474357000	-0.585884000	-0.216249000
6	-6.017498000	-1.116279000	0.996708000
6	-7.734004000	-0.953871000	-0.701281000
1	-5.055391000	-0.824000000	1.403457000
1	-8.100850000	-0.509880000	-1.619821000
6	-6.821423000	-2.004641000	1.714898000
6	-8.520135000	-1.853989000	0.011380000
1	-6.467902000	-2.399786000	2.659951000
1	-9.497874000	-2.126787000	-0.367643000
6	-8.067257000	-2.380345000	1.221890000
1	-8.688292000	-3.069182000	1.781461000
6	-5.035892000	-0.294550000	-2.118743000
1	-4.597347000	0.445807000	-2.789053000
1	-5.719389000	-0.932621000	-2.677251000
1	4.654316000	2.837888000	-2.501292000
1	-4.309465000	-1.876442000	-1.224488000
1	4.666484000	0.977128000	0.471852000

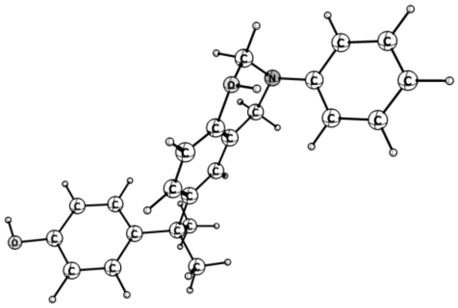

O-Monobz

6	5.963128000	-1.356561000	-0.471972000
6	4.742850000	-1.639515000	-1.090974000
6	6.117362000	-0.155600000	0.217224000
1	4.608996000	-2.572386000	-1.630583000
6	3.699642000	-0.723230000	-1.015786000
6	5.062617000	0.750969000	0.281787000
1	2.761737000	-0.970947000	-1.502023000
1	5.223474000	1.674367000	0.822887000
6	3.831777000	0.492125000	-0.331669000
6	2.656755000	1.489913000	-0.315999000
6	1.406224000	0.797965000	0.260007000
6	0.149050000	0.885982000	-0.334403000
6	1.507183000	0.077769000	1.464968000
1	0.028043000	1.418982000	-1.269984000
1	2.475790000	-0.028538000	1.938218000
6	-0.982662000	0.292425000	0.244212000

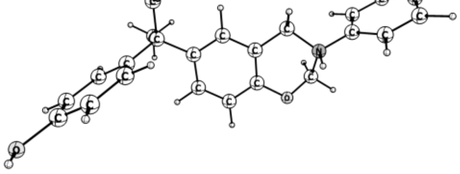

N-Monobz

6	0.403982000	-0.513926000	2.058618000
1	0.487345000	-1.069708000	2.984464000
6	-0.837158000	-0.393047000	1.444186000
6	2.940033000	2.721986000	0.577771000
1	2.070878000	3.384196000	0.581031000
1	3.150116000	2.442046000	1.612153000
1	3.789953000	3.292226000	0.196221000
6	2.433686000	2.002920000	-1.757535000
1	2.152472000	1.199504000	-2.442255000
1	1.667078000	2.782635000	-1.797545000
1	3.362191000	2.439009000	-2.129940000
8	7.028127000	-2.207609000	-0.503279000
1	6.818345000	-2.989480000	-1.025069000
8	-1.927263000	-1.040439000	2.038030000
6	-2.326251000	0.412298000	-0.435541000
1	-2.753106000	1.412058000	-0.341757000
1	-2.279383000	0.164745000	-1.497556000
7	-3.326540000	-0.542650000	0.192147000
6	-3.154698000	-0.505993000	1.723881000
1	-3.933720000	-1.128218000	2.158732000
1	-3.262578000	0.541594000	2.018538000
6	-4.725253000	-0.356839000	-0.256871000
6	-5.347069000	-1.413980000	-0.912089000
6	-5.386027000	0.840570000	-0.000212000
1	-4.817886000	-2.342658000	-1.102793000
1	-4.898806000	1.661730000	0.511906000
6	-6.668669000	-1.265042000	-1.327594000
6	-6.708589000	0.973622000	-0.418064000
1	-7.162192000	-2.080009000	-1.842269000
1	-7.237013000	1.898866000	-0.224466000
6	-7.347374000	-0.073775000	-1.080339000
1	-8.374879000	0.039407000	-1.403633000
1	7.065639000	0.058807000	0.694754000
1	-3.046852000	-1.491516000	-0.073695000

6	6.110150000	-0.656892000	-0.912291000
6	4.922642000	-0.846119000	-1.623918000
6	6.112784000	0.178710000	0.202469000
1	4.906140000	-1.495878000	-2.493892000
6	3.760797000	-0.200146000	-1.216438000
6	4.940501000	0.818977000	0.595846000
1	2.852027000	-0.370229000	-1.784321000
1	4.985265000	1.463435000	1.464215000
6	3.739234000	0.647611000	-0.101150000
6	2.432737000	1.372098000	0.280060000
6	1.312909000	0.332729000	0.484953000
6	0.038489000	0.459930000	-0.065721000
6	1.547618000	-0.791137000	1.293929000
1	-0.189069000	1.297941000	-0.714142000
1	2.531981000	-0.937377000	1.723015000
6	-0.973456000	-0.485215000	0.167554000
6	0.563531000	-1.737314000	1.548771000
1	0.782532000	-2.595699000	2.176241000
6	-0.702087000	-1.589067000	0.984506000
6	2.564608000	2.167173000	1.601461000
1	1.610049000	2.641225000	1.843899000
1	2.843049000	1.527380000	2.441682000
1	3.312245000	2.958295000	1.509921000
6	2.093779000	2.383788000	-0.839239000
1	1.912633000	1.892027000	-1.797618000
1	1.219417000	2.991662000	-0.586667000
1	2.936115000	3.064295000	-0.975128000
8	7.285429000	-1.258730000	-1.253021000
1	7.172649000	-1.789577000	-2.048717000
8	-1.729665000	-2.471848000	1.194901000
6	-2.309292000	-0.365119000	-0.489566000
1	-2.295894000	0.338246000	-1.324063000

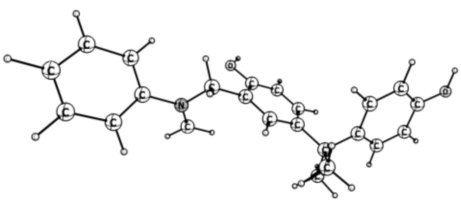

ROP-MonoBz

1	-2.667020000	-1.325555000	-0.860089000
7	-3.423064000	0.134623000	0.419322000
6	-3.200911000	0.563290000	1.601952000
1	-4.032863000	0.893031000	2.212362000
1	-2.181929000	0.583693000	1.971514000
6	-4.761298000	0.150743000	-0.145683000
6	-5.325017000	-1.039728000	-0.602789000
6	-5.445442000	1.363471000	-0.219204000
1	-4.790126000	-1.977528000	-0.517139000
1	-4.975399000	2.282185000	0.112580000
6	-6.612189000	-1.007679000	-1.131059000
6	-6.729165000	1.378769000	-0.758433000
1	-7.069995000	-1.927275000	-1.474139000
1	-7.267429000	2.315852000	-0.829745000
6	-7.311372000	0.196648000	-1.211884000
1	-8.310120000	0.213199000	-1.630861000
1	7.036163000	0.322173000	0.750230000
1	-1.422379000	-3.236373000	1.695812000

6	-3.218174000	-1.624433000	-1.583531000
6	-1.892632000	-1.890576000	-1.927475000
6	-3.549252000	-1.256223000	-0.274532000
1	-1.644916000	-2.176231000	-2.945636000
6	-0.891401000	-1.788651000	-0.969219000
6	-2.518545000	-1.162364000	0.663964000
1	0.131473000	-1.994567000	-1.265867000
1	-2.794291000	-0.874194000	1.670528000
6	-1.181342000	-1.422543000	0.350543000
6	-0.043988000	-1.338765000	1.388340000
6	0.991916000	-0.293861000	0.927029000
6	2.369834000	-0.508541000	0.988534000
6	0.557972000	0.962834000	0.478391000
1	2.754318000	-1.460798000	1.333637000
1	-0.504567000	1.160061000	0.399691000
6	3.291526000	0.486600000	0.636155000
6	1.449897000	1.963981000	0.111552000
1	1.081018000	2.922674000	-0.239299000
6	2.815547000	1.724160000	0.195602000
6	-0.546869000	-0.888949000	2.781693000
1	0.294932000	-0.828323000	3.475541000
1	-1.023091000	0.093209000	2.751828000
1	-1.262483000	-1.607191000	3.188619000
6	0.575239000	-2.746329000	1.552138000
1	1.034550000	-3.107022000	0.628944000
1	1.325933000	-2.768390000	2.347456000
1	-0.209246000	-3.453969000	1.825842000
8	-4.241136000	-1.692569000	-2.491993000
8	3.773915000	2.657647000	-0.168487000
6	-4.983650000	-0.972195000	0.110572000
1	-5.616329000	-1.832956000	-0.156867000
1	-5.030493000	-0.862085000	1.195679000
7	-5.463369000	0.262642000	-0.505229000
6	-6.746997000	0.749072000	-0.221817000
6	-7.299479000	1.731080000	-1.064887000
6	-7.491450000	0.334964000	0.893018000
1	-6.735482000	2.063825000	-1.931098000
1	-7.111298000	-0.429673000	1.558136000
6	-8.545280000	2.280489000	-0.796722000
6	-8.740040000	0.899795000	1.156183000
1	-8.948212000	3.033986000	-1.464615000
1	-9.296829000	0.563040000	2.023869000
6	-9.278078000	1.872695000	0.320418000
1	-10.250158000	2.302849000	0.528547000
6	4.768211000	0.265050000	0.773193000
1	5.238979000	0.960329000	1.470081000
1	5.010119000	-0.753634000	1.071831000
7	5.482645000	0.516191000	-0.566456000

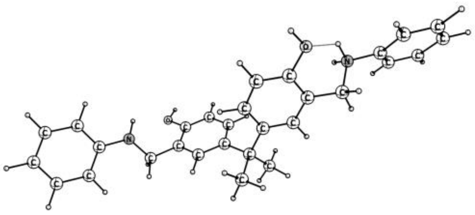

PA-BPA-PA

6	6.951688000	0.361908000	-0.544340000
6	7.502908000	-0.874473000	-0.857704000
6	7.733532000	1.452549000	-0.183059000
1	6.874556000	-1.712664000	-1.141439000
1	7.281554000	2.409770000	0.053825000
6	8.887903000	-1.018943000	-0.808753000
6	9.116994000	1.293182000	-0.136989000
1	9.335231000	-1.973597000	-1.056229000
1	9.742728000	2.133405000	0.137333000
6	9.691328000	0.061500000	-0.447753000
1	10.767579000	-0.055369000	-0.413039000
1	3.365938000	3.504077000	-0.386398000
1	5.074973000	-0.105534000	-1.269147000
1	-3.919787000	-2.020207000	-3.338823000
1	-5.245940000	0.304109000	-1.491678000
1	5.218149000	1.474520000	-0.838881000

6	3.291424000	0.068951000	-1.343990000
6	2.053790000	0.703025000	-1.496711000
6	3.515894000	-0.765098000	-0.241406000
1	1.905841000	1.353277000	-2.350794000
6	1.047419000	0.492819000	-0.568411000
6	2.479441000	-0.960523000	0.677818000
1	0.095307000	0.992744000	-0.713472000
1	2.680312000	-1.603860000	1.527101000
6	1.233784000	-0.347854000	0.541416000
6	0.099801000	-0.506609000	1.573409000
6	-1.187193000	-0.953153000	0.852498000
6	-2.431041000	-0.359504000	1.071603000
6	-1.142302000	-2.036522000	-0.038080000
1	-2.518196000	0.477921000	1.753262000
1	-0.189960000	-2.507959000	-0.249652000
6	-3.595363000	-0.824640000	0.445231000
6	-2.279321000	-2.512761000	-0.680228000
1	-2.208427000	-3.346412000	-1.371807000
6	-3.504780000	-1.906119000	-0.434320000
6	0.415483000	-1.580133000	2.642914000
1	-0.431845000	-1.684127000	3.324880000
1	0.608714000	-2.559091000	2.198930000
1	1.285668000	-1.294389000	3.238145000
6	-0.079460000	0.842814000	2.307401000
1	-0.376998000	1.644392000	1.627320000
1	-0.816293000	0.772845000	3.113389000
1	0.870842000	1.134282000	2.757867000
8	4.243768000	0.316704000	-2.286994000
8	-4.683501000	-2.298258000	-1.050492000
6	4.877678000	-1.414005000	-0.055907000
1	5.173615000	-1.369637000	0.991490000
1	4.840544000	-2.474551000	-0.335286000
7	5.893003000	-0.738469000	-0.875109000
6	6.697811000	0.267571000	-0.265442000
6	6.793401000	1.567223000	-0.779111000
6	7.460141000	-0.068367000	0.863357000
1	6.192157000	1.864064000	-1.629031000
1	7.427348000	-1.080777000	1.249666000
6	7.644232000	2.498275000	-0.183160000
6	8.287062000	0.873183000	1.465318000
1	7.707683000	3.497926000	-0.598412000
1	8.872198000	0.590240000	2.333176000
6	8.389387000	2.162891000	0.943071000
1	9.043536000	2.891842000	1.406275000
6	5.416775000	-0.544174000	-2.201542000
1	5.133479000	-1.512956000	-2.625959000
1	6.165990000	-0.069833000	-2.829533000
6	-4.933258000	-0.212516000	0.733090000
1	-5.645334000	-0.923725000	1.154634000
1	-4.862533000	0.651280000	1.392059000

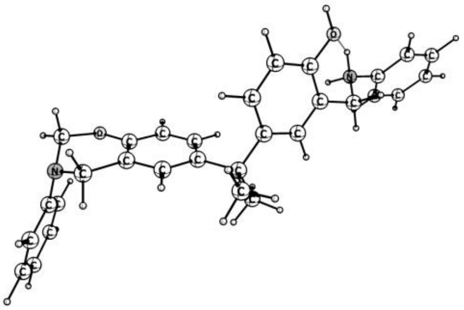

NH-PA-MonoBz

7	-5.596316000	0.275202000	-0.567631000	
6	-6.937767000	0.874289000	-0.414464000	
6	-8.048905000	0.039739000	-0.428543000	
6	-7.044115000	2.247829000	-0.233463000	
1	-7.942224000	-1.029706000	-0.575475000	
1	-6.163743000	2.882507000	-0.229920000	
6	-9.309566000	0.607495000	-0.255765000	
6	-8.311162000	2.801989000	-0.063289000	
1	-10.187448000	-0.026475000	-0.269187000	
1	-8.413359000	3.871505000	0.072359000	
6	-9.439441000	1.983418000	-0.072842000	
1	-10.422350000	2.419302000	0.057848000	
1	-4.546807000	-3.090315000	-1.583611000	
1	-5.631703000	-0.552775000	-1.180408000	
1	-4.964060000	0.940575000	-1.019492000	
6	2.678188000	-1.930897000	-0.027737000	
6	1.306878000	-2.151188000	0.086975000	
6	3.147337000	-0.726533000	-0.565673000	
1	0.938573000	-3.082801000	0.505149000	
6	0.414347000	-1.174490000	-0.334525000	
6	2.220562000	0.242961000	-0.981311000	
1	-0.647842000	-1.360773000	-0.228136000	
1	2.604162000	1.161403000	-1.409711000	
6	0.844294000	0.045807000	-0.880399000	
6	-0.195231000	1.056983000	-1.403003000	
6	-1.301680000	1.252924000	-0.347222000	
6	-2.662099000	1.091909000	-0.627828000	
6	-0.962423000	1.659745000	0.948601000	
1	-2.981102000	0.789486000	-1.618150000	
1	0.079217000	1.798973000	1.218656000	
6	-3.664626000	1.318549000	0.319650000	
6	-1.934654000	1.898790000	1.911970000	
1	-1.645094000	2.222515000	2.907511000	
6	-3.287116000	1.730759000	1.604829000	
6	0.433178000	2.437306000	-1.706602000	
1	-0.351123000	3.127534000	-2.022086000	
1	0.917505000	2.871299000	-0.828618000	
1	1.164148000	2.380708000	-2.518666000	
6	-0.743853000	0.492640000	-2.736103000	
1	-1.246964000	-0.467273000	-2.601205000	
1	-1.448637000	1.188980000	-3.196158000	
1	0.078622000	0.340886000	-3.439045000	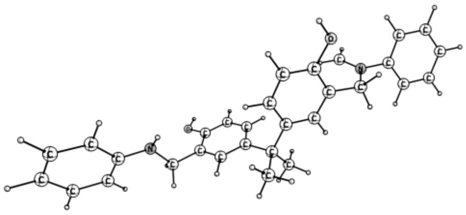
8	3.623626000	-2.832097000	0.390387000	
8	-4.276280000	1.964755000	2.511743000	**ROP-PA-MonoBz**
6	4.614546000	-0.526727000	-0.768342000	
1	5.101482000	-1.424501000	-1.153895000	
1	4.814245000	0.292740000	-1.458674000	
7	5.402879000	-0.197351000	0.489910000	
6	6.841575000	-0.064858000	0.333331000	
6	7.675865000	-0.915137000	1.057699000	
6	7.351693000	0.895516000	-0.538991000	
1	7.255977000	-1.680667000	1.699813000	
1	6.694183000	1.560972000	-1.084764000	
6	9.054785000	-0.788850000	0.910401000	
6	8.732309000	1.013641000	-0.668666000	
1	9.713246000	-1.449396000	1.460922000	
1	9.142939000	1.766738000	-1.329831000	
6	9.581700000	0.173599000	0.051436000	
1	10.654965000	0.267844000	-0.060427000	
6	4.855697000	-0.114834000	1.641242000	
1	3.788366000	-0.278574000	1.731287000	
1	5.467124000	0.124730000	2.502815000	
6	-5.118511000	1.109771000	-0.033564000	
1	-5.726901000	1.841249000	0.503018000	
1	-5.259335000	1.291820000	-1.110354000	
7	-5.551356000	-0.229451000	0.349310000	

6	-6.846664000	-0.682274000	0.093711000
6	-7.125730000	-2.057853000	0.198153000
6	-7.898186000	0.188524000	-0.231893000
1	-6.324622000	-2.744416000	0.456075000
1	-7.722363000	1.252608000	-0.324002000
6	-8.410022000	-2.540083000	-0.011863000
6	-9.185572000	-0.308656000	-0.435156000
1	-8.598186000	-3.604633000	0.075233000
1	-9.982868000	0.383541000	-0.682930000
6	-9.455136000	-1.669426000	-0.329310000
1	-10.457103000	-2.047709000	-0.491046000
1	-3.902967000	2.264240000	3.347340000
1	-4.839005000	-0.937168000	0.247610000
1	3.211228000	-3.669722000	0.631823000

6	2.767439000	-1.258239000	-0.275239000
6	1.430790000	-1.615119000	-0.239734000
6	3.174296000	0.029768000	-0.595056000
1	1.137094000	-2.627429000	0.008874000
6	0.489017000	-0.633280000	-0.535245000
6	2.214069000	1.005702000	-0.872797000
1	-0.561744000	-0.895279000	-0.500882000
1	2.540544000	2.007692000	-1.122840000
6	0.852002000	0.687144000	-0.853969000
6	-0.246112000	1.701935000	-1.232280000
6	-1.393876000	1.618209000	-0.206127000
6	-2.707989000	1.297424000	-0.558577000
6	-1.138657000	1.886502000	1.143923000
1	-2.956250000	1.080485000	-1.589873000
1	-0.134525000	2.134981000	1.470575000
6	-3.749337000	1.239830000	0.370914000
6	-2.151370000	1.836976000	2.094190000
1	-1.931067000	2.049150000	3.136320000
6	-3.453311000	1.513801000	1.711161000
6	0.281014000	3.155356000	-1.262086000
1	-0.546582000	3.832083000	-1.481468000
1	0.709390000	3.461164000	-0.304906000
1	1.033881000	3.297312000	-2.042781000
6	-0.707528000	1.346969000	-2.667044000
1	-1.118750000	0.337619000	-2.731728000
1	-1.465411000	2.051788000	-3.016690000
1	0.141344000	1.404052000	-3.352522000
8	3.790794000	-2.178502000	-0.011744000
8	-4.485057000	1.434497000	2.608411000
6	4.654944000	0.228857000	-0.700582000
1	5.074451000	-0.300714000	-1.556388000
1	4.958263000	1.274072000	-0.749354000
7	5.354100000	-0.379100000	0.532408000
6	6.827109000	-0.456841000	0.390749000
6	7.385314000	-1.339358000	-0.529737000
6	7.611470000	0.370439000	1.185441000
1	6.764586000	-1.985495000	-1.140008000
1	7.160245000	1.052252000	1.900007000
6	8.771801000	-1.385401000	-0.650078000
6	8.998517000	0.313879000	1.053882000
1	9.221949000	-2.067865000	-1.360363000
1	9.620487000	0.952375000	1.668814000
6	9.576053000	-0.561311000	0.137731000
1	10.653660000	-0.604443000	0.037594000
6	4.671929000	-1.735623000	0.950967000
1	4.175924000	-1.523260000	1.901969000
1	5.465258000	-2.467894000	1.066085000
6	-5.158527000	0.895856000	-0.056121000
1	-5.849543000	1.685902000	0.277145000
1	-5.190099000	0.878797000	-1.147212000
7	-5.558340000	-0.417438000	0.443238000
6	-6.805012000	-0.960335000	0.102865000

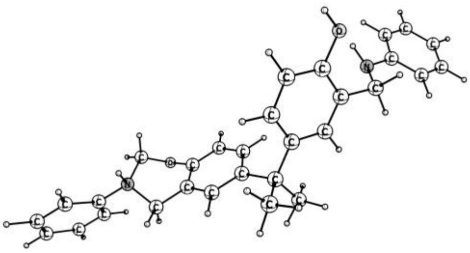

N-PA-MonoBz

6	-7.297324000	-2.045348000	0.851616000
6	-7.567271000	-0.500888000	-0.981735000
1	-6.718556000	-2.414414000	1.693005000
1	-7.233602000	0.341275000	-1.574185000
6	-8.502350000	-2.649390000	0.521737000
6	-8.774109000	-1.120869000	-1.307982000
1	-8.859306000	-3.482412000	1.117418000
1	-9.345785000	-0.747490000	-2.150638000
6	-9.252686000	-2.195151000	-0.565420000
1	-10.192850000	-2.668044000	-0.821850000
1	-4.192953000	1.707887000	3.484581000
1	-5.344857000	-0.531494000	1.424805000
1	5.166757000	0.266180000	1.301419000

6	2.943421000	-0.375125000	0.905204000
6	1.661618000	-0.888624000	0.922726000
6	3.354423000	0.754112000	0.225930000
1	1.410134000	-1.770189000	1.499771000
6	0.710296000	-0.206444000	0.176959000
6	2.356808000	1.414262000	-0.505528000
1	-0.311070000	-0.566094000	0.178125000
1	2.638370000	2.308604000	-1.047652000
6	1.038584000	0.952572000	-0.552682000
6	-0.050450000	1.630673000	-1.406993000
6	-1.330434000	1.777744000	-0.559956000
6	-2.539863000	1.162685000	-0.896811000
6	-1.307782000	2.561330000	0.600175000
1	-2.608756000	0.547645000	-1.785281000
1	-0.393993000	3.057917000	0.908389000
6	-3.699135000	1.302932000	-0.130620000
6	-2.443596000	2.721143000	1.384466000
1	-2.404148000	3.334687000	2.279592000
6	-3.636135000	2.093342000	1.023325000
6	0.378445000	3.027399000	-1.911445000
1	-0.449519000	3.476423000	-2.462341000
1	0.633602000	3.705512000	-1.094466000
1	1.230662000	2.968109000	-2.594357000
6	-0.260476000	0.732158000	-2.651086000
1	-0.582586000	-0.277115000	-2.387035000
1	-1.007631000	1.168522000	-3.317520000
1	0.674877000	0.648629000	-3.209645000
8	3.975013000	-1.091849000	1.716869000
8	-4.778565000	2.201107000	1.768600000
6	4.782913000	1.262542000	0.329547000
1	5.190439000	1.476619000	-0.658275000
1	4.785620000	2.207617000	0.884423000
7	5.678228000	0.318006000	1.017118000
6	6.466734000	-0.595062000	0.217645000
6	6.008289000	-1.119489000	-0.997273000
6	7.723493000	-0.971988000	0.703342000
1	5.048328000	-0.820809000	-1.404402000
1	8.091859000	-0.532718000	1.623549000
6	6.807791000	-2.010729000	-1.716832000
6	8.505057000	-1.875024000	-0.010669000
1	6.452982000	-2.401352000	-2.663275000
1	9.480555000	-2.154874000	0.368987000
6	8.050607000	-2.395392000	-1.223170000
1	8.668121000	-3.086610000	-1.783698000
6	5.028524000	-0.299997000	2.119904000
1	4.593994000	0.441450000	2.791646000
1	5.708000000	-0.943286000	2.677313000
6	-4.987655000	0.621520000	-0.532923000
1	-5.796937000	1.365410000	-0.595579000
1	-4.855110000	0.203698000	-1.532578000
7	-5.319221000	-0.464428000	0.386550000
6	-6.430309000	-1.286893000	0.147063000
6	-6.916345000	-2.087158000	1.197303000

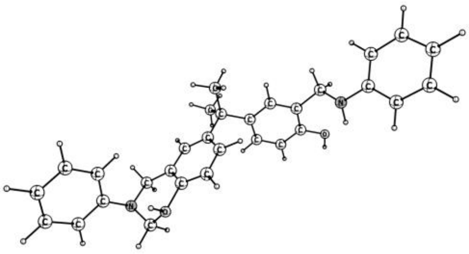

O-PA-MonoBz

6	-7.052969000	-1.384156000	-1.106175000
1	-6.444916000	-2.024189000	2.173495000
1	-6.722820000	-0.773495000	-1.936641000
6	-7.980076000	-2.955611000	0.996726000
6	-8.117292000	-2.266235000	-1.297681000
1	-8.336703000	-3.558639000	1.824541000
1	-8.583430000	-2.323079000	-2.275276000
6	-8.589866000	-3.057187000	-0.255839000
1	-9.420064000	-3.735421000	-0.410790000
1	-4.653142000	2.821030000	2.495253000
1	-5.261705000	-0.171969000	1.352780000
1	4.292622000	-1.876947000	1.223736000

6	3.268621000	-0.576845000	0.814009000
6	2.040544000	-1.199260000	0.704693000
6	3.600558000	0.656849000	0.291560000
1	1.848020000	-2.160937000	1.164520000
6	1.061832000	-0.518077000	-0.005404000
6	2.577169000	1.309722000	-0.410365000
1	0.077524000	-0.960045000	-0.095405000
1	2.795935000	2.284552000	-0.828375000
6	1.310097000	0.744758000	-0.577268000
6	0.193270000	1.428519000	-1.389266000
6	-1.113729000	1.388835000	-0.573569000
6	-2.296389000	0.829760000	-1.056884000
6	-1.141563000	1.955566000	0.712043000
1	-2.322704000	0.369667000	-2.038091000
1	-0.243126000	2.397241000	1.130475000
6	-3.480216000	0.825962000	-0.310884000
6	-2.300381000	1.970942000	1.469873000
1	-2.322496000	2.414930000	2.457848000
6	-3.476438000	1.413585000	0.957879000
6	0.522977000	2.902424000	-1.719468000
1	-0.321700000	3.344996000	-2.249817000
1	0.692369000	3.499367000	-0.820829000
1	1.398809000	2.988842000	-2.369293000
6	0.084617000	0.662092000	-2.730332000
1	-0.183726000	-0.386983000	-2.588628000
1	-0.664137000	1.124145000	-3.377216000
1	1.041744000	0.695342000	-3.256436000
8	4.326234000	-1.289709000	1.595340000
8	-4.589369000	1.459733000	1.740776000
6	4.968271000	1.274617000	0.530517000
1	5.397461000	1.638881000	-0.402639000
1	4.856486000	2.144315000	1.187890000
7	5.917335000	0.339221000	1.155697000
6	6.825340000	-0.398212000	0.303983000
6	6.477070000	-0.810291000	-0.988343000
6	8.087571000	-0.718897000	0.815374000
1	5.513524000	-0.550614000	-1.413291000
1	8.368650000	-0.363185000	1.800212000
6	7.390685000	-1.533860000	-1.758398000
6	8.984154000	-1.456493000	0.048470000
1	7.119994000	-1.836728000	-2.763012000
1	9.962527000	-1.692993000	0.449613000
6	8.639361000	-1.864318000	-1.240830000
1	9.345587000	-2.425286000	-1.840720000
6	5.280259000	-0.459590000	2.143755000
1	4.746808000	0.155919000	2.869232000
1	5.991789000	-1.100639000	2.662390000
6	-4.736252000	0.165667000	-0.845176000
1	-5.157568000	0.766691000	-1.671399000
1	-4.484234000	-0.815325000	-1.255058000
7	-5.681624000	-0.015080000	0.245600000
6	-5.805640000	1.182266000	1.038382000
1	-6.575507000	1.075096000	1.794315000
1	-6.028995000	2.060521000	0.409629000

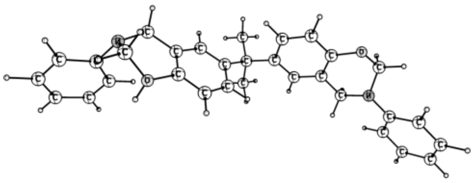

O-BisBz

6	-6.813526000	-0.844626000	0.064730000
6	-7.574934000	-1.242123000	1.177718000
6	-7.180390000	-1.324932000	-1.200250000
1	-7.287985000	-0.920301000	2.171848000
1	-6.625218000	-1.032243000	-2.082087000
6	-8.674323000	-2.076236000	1.020981000
6	-8.276332000	-2.176643000	-1.344007000
1	-9.244406000	-2.370550000	1.894992000
1	-8.540451000	-2.534963000	-2.332717000
6	-9.034124000	-2.552939000	-0.240807000
1	-9.888161000	-3.209022000	-0.357121000
1	4.739334000	-1.979199000	1.034855000

6	3.108937000	-0.844037000	-1.158623000
6	1.800569000	-1.148866000	-1.491683000
6	3.456358000	0.374043000	-0.591302000
1	1.553352000	-2.105684000	-1.934605000
6	0.825857000	-0.189079000	-1.234204000
6	2.462008000	1.319522000	-0.327952000
1	-0.205553000	-0.419872000	-1.472341000
1	2.741966000	2.266930000	0.115941000
6	1.127276000	1.054240000	-0.650970000
6	-0.004074000	2.080218000	-0.438713000
6	-1.189777000	1.388740000	0.262588000
6	-2.483407000	1.380723000	-0.257569000
6	-0.984550000	0.748672000	1.496481000
1	-2.690270000	1.848652000	-1.213259000
1	0.008077000	0.722700000	1.933808000
6	-3.553191000	0.769936000	0.406905000
6	-2.025813000	0.143698000	2.180180000
1	-1.867285000	-0.342950000	3.135146000
6	-3.316092000	0.159512000	1.641571000
6	0.444992000	3.277359000	0.430972000
1	-0.404262000	3.944869000	0.586485000
1	0.797259000	2.962788000	1.415940000
1	1.233857000	3.859181000	-0.055075000
6	-0.378754000	2.634693000	-1.834437000
1	-0.744281000	1.854068000	-2.504828000
1	-1.147564000	3.406344000	-1.752183000
1	0.498823000	3.089341000	-2.300242000
8	4.163923000	-1.737375000	-1.388413000
8	-4.306863000	-0.445314000	2.356171000
6	4.923456000	0.589182000	-0.381832000
1	5.460182000	0.678135000	-1.326721000
1	5.159584000	1.451036000	0.241272000
7	5.534385000	-0.643270000	0.315743000
6	7.015473000	-0.623239000	0.350136000
6	7.734595000	-0.765402000	-0.833229000
6	7.645183000	-0.451606000	1.577010000
1	7.233788000	-0.905562000	-1.784406000
1	7.069548000	-0.343745000	2.491378000
6	9.125401000	-0.732227000	-0.774228000
6	9.038529000	-0.419973000	1.620994000
1	9.699550000	-0.843158000	-1.685792000
1	9.540653000	-0.288602000	2.571405000
6	9.775699000	-0.559093000	0.447934000
1	10.857935000	-0.535168000	0.484232000
6	4.917959000	-1.974174000	-0.259088000
1	4.322581000	-2.388613000	0.558961000
1	5.748757000	-2.622614000	-0.520066000
6	-4.935976000	0.732480000	-0.214073000
1	-5.384401000	1.742516000	-0.201939000
1	-4.854879000	0.427324000	-1.260092000
7	-5.752036000	-0.242912000	0.492578000
6	-5.625633000	-0.100373000	1.921207000
1	-6.302783000	-0.766638000	2.443916000
1	-5.815496000	0.938624000	2.239504000

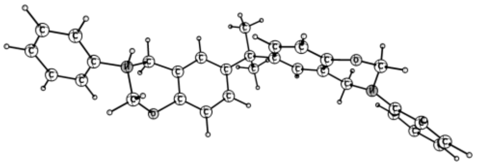

N-BisBz

6	-6.987160000	-0.657560000	-0.056737000
6	-7.647354000	-1.781616000	0.470371000
6	-7.562253000	-0.001198000	-1.154303000
1	-7.203736000	-2.334064000	1.290205000
1	-7.089097000	0.873571000	-1.581253000
6	-8.848415000	-2.216825000	-0.074555000
6	-8.760454000	-0.457512000	-1.704730000
1	-9.336477000	-3.087901000	0.348083000
1	-9.184820000	0.066902000	-2.553646000
6	-9.415479000	-1.560327000	-1.168275000
1	-10.348643000	-1.908028000	-1.594524000
1	5.217264000	-0.595055000	1.285304000

6	-3.020696000	-0.824863000	1.879299000
6	-1.662648000	-0.951030000	2.166238000
6	-3.460270000	0.194683000	1.026833000
1	-1.316434000	-1.742470000	2.823649000
6	-0.754260000	-0.064917000	1.603191000
6	-2.518029000	1.077017000	0.473631000
1	0.299011000	-0.189970000	1.825470000
1	-2.880809000	1.868061000	-0.172036000
6	-1.155242000	0.972273000	0.745178000
6	-0.102854000	1.958879000	0.201243000
6	1.076865000	1.163528000	-0.392276000
6	2.398408000	1.341414000	0.015152000
6	0.835254000	0.223250000	-1.408011000
1	2.635547000	2.044735000	0.805167000
1	-0.179333000	0.046385000	-1.749378000
6	3.459715000	0.627026000	-0.552822000
6	1.866397000	-0.491237000	-1.994591000
1	1.678862000	-1.210004000	-2.783403000
6	3.184077000	-0.285802000	-1.574360000
6	-0.676348000	2.875498000	-0.904176000
1	0.118355000	3.519801000	-1.284306000
1	-1.067780000	2.306235000	-1.750447000
1	-1.469244000	3.524879000	-0.520986000
6	0.325285000	2.865856000	1.379699000
1	0.776418000	2.297169000	2.195607000
1	1.041284000	3.622522000	1.050494000
1	-0.547300000	3.386781000	1.780925000
8	-3.976144000	-1.678833000	2.368269000
1	-3.596356000	-2.270243000	3.028496000
8	4.163474000	-1.010310000	-2.186524000
6	-4.922594000	0.373337000	0.777987000
1	-5.513894000	0.241452000	1.686027000
1	-5.141130000	1.359319000	0.367979000
7	-5.534638000	-0.617001000	-0.201868000
6	-6.968692000	-0.513556000	-0.412478000
6	-7.771501000	-1.613250000	-0.112830000
6	-7.507286000	0.677846000	-0.896072000
1	-7.335128000	-2.515737000	0.299160000
1	-6.871829000	1.520270000	-1.140020000
6	-9.145821000	-1.516668000	-0.315364000
6	-8.881830000	0.755094000	-1.099936000
1	-9.781064000	-2.361188000	-0.078247000
1	-9.311989000	1.668718000	-1.491311000
6	-9.699354000	-0.336998000	-0.809026000
1	-10.768987000	-0.266233000	-0.964525000
6	-4.852651000	-1.542724000	-0.758462000
1	-3.796094000	-1.628331000	-0.533222000
1	-5.341328000	-2.218453000	-1.449955000
6	4.877708000	0.806397000	-0.046643000
1	5.263249000	1.798685000	-0.343456000
1	4.879217000	0.772527000	1.045475000
7	5.706178000	-0.280221000	-0.546427000
6	5.483247000	-0.509968000	-1.951767000
1	6.166007000	-1.257024000	-2.340875000

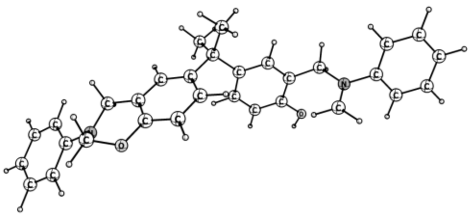

ROP-BisBz

1	5.589848000	0.421718000	-2.532704000	
6	6.995916000	-0.487976000	-0.005667000	
6	7.690192000	-1.678584000	-0.284448000	
6	7.596429000	0.448946000	0.847494000	
1	7.231620000	-2.439258000	-0.905090000	
1	7.097824000	1.381519000	1.077904000	
6	8.947155000	-1.908754000	0.259571000	
6	8.852209000	0.199648000	1.402237000	
1	9.460774000	-2.836002000	0.031501000	
1	9.295212000	0.939691000	2.059384000	
6	9.539423000	-0.972964000	1.109448000	
1	10.516835000	-1.160262000	1.537185000	
